# Protocol for the Houston Hospital-based violence intervention program

**DOI:** 10.1371/journal.pone.0325569

**Published:** 2025-07-02

**Authors:** Alexander Testa, Eresha Bluth, Latanya Monroe, Karlton Harris, Sarah Beth Abbott, Mary E. Aitken, Erin E. Fox, Brian Heckler, Lillian S. Kao, Ruosha Li, Susannah (Meg) Michael, Heidi M. McPherson, Marisol Nieves, Christian P. Owen, Kevin Rix, Hema Sarvani Jalaparthi, Vanessa Schick, Shreela V. Sharma, Carlie Stratemann, Anne Marie V. Thompson, Jack Tsai, Zixi Yang, Sandra McKay

**Affiliations:** 1 Department of Management, Policy and Community Health, School of Public Health, University of Texas Health Science Center at Houston, Houston, Texas, United States of America; 2 Department of Pediatrics, McGovern Medical School, University of Texas Health Science Center at Houston, Houston, Texas, United States of America; 3 The Forgotten Third, Houston, Texas, United States of America; 4 Children’s Memorial Hermann Hospital, Houston, Texas, United States of America; 5 Department of Surgery, McGovern Medical School, University of Texas Health Science Center at Houston, Houston, Texas, United States of America; 6 Department of Biostatistics and Data Science, School of Public Health, University of Texas Health Science Center at Houston, Houston, Texas, United States of America; 7 Memorial Hermann Hospital, Houston, Texas, United States of America; 8 Health Equity Collective, School of Public Health, University of Texas Health Science Center at Houston, Houston, Texas, United States of America; 9 Department of Research, Cizik School of Nursing, University of Texas Health Science Center at Houston, Houston, Texas, United States of America; 10 Department of Health Promotion & Behavioral Sciences, School of Public Health, University of Texas Health Science Center at Houston, Houston, Texas, United States of America; 11 Department of Epidemiology, School of Public Health, University of Texas Health Science Center at Houston, Houston, Texas, United States of America; 12 Michael & Susan Dell Center for Healthy Living, School of Public Health, University of Texas Health Science Center at Houston, Houston, Texas, United States of America; 13 National Center on Homelessness Among Veterans, United States of America Department of Veterans Affairs, Washington, District of Columbia, United States of America; 14 Department of Psychiatry, Yale University School of Medicine, New Haven, Connecticut, United States of America; PLOS: Public Library of Science, UNITED KINGDOM OF GREAT BRITAIN AND NORTHERN IRELAND

## Abstract

Firearm violence is a leading cause of injury and mortality in the United States. Hospital-based violence intervention programs (HVIPs) are a promising public health strategy designed to reduce recurrent violence by engaging patients during hospitalization and connecting them to support services after discharge. This protocol describes the design and implementation of the Houston Hospital-Based Violence Intervention Program (Houston-HVIP), which will be evaluated by a randomized controlled trial conducted at a Level 1 trauma center in Houston, Texas. The study plans to enroll individuals aged 16–35 who present with gunshot wounds (GSW) at the Level 1 trauma center. Participants are randomized to either a treatment group receiving six months of intensive case management with direct referrals to social services or a control group receiving usual care, which involves indirect referral and limited case management. The primary outcome is a composite measure of an individual’s exposure to firearm violence via (a) self-report, (b) hospital admission records, and (c) mortality records. Secondary outcomes measured at the individual level assess violent reinjury, attitudes toward violence, post-traumatic stress, aggression, and self-rated health. Outcomes are assessed at baseline and 3-, 6-, 9-, and 12-months post-enrollment. The study will enroll 274 participants and include both quantitative and qualitative assessments to evaluate program impact and participant experience. This protocol aims to contribute to the design and implementation of HVIPs in large Level 1 trauma centers.

## Introduction

Firearm violence is a critical public health crisis in the United States (US), resulting in approximately 46,000 deaths in 2023 [1], as well as tens of thousands of additional individuals who are admitted to the hospital for non-fatal gunshot wounds (GSW) annually [2,3]. GSWs result in long-term physical, functional, psychological, and social consequences [4], as well as an economic cost estimated at approximately $500 billion annually [5], with the median medical charge for a single GSW estimated at $53,832 [6]. Accordingly, interventions aimed at reducing firearm violence and hospital admission for GSWs are essential components of a comprehensive public health response.

Hospital-based violence intervention programs (HVIPs) are a novel strategy with the potential to mitigate firearm violence [7–9]. HVIPs operate within trauma centers and aim to reduce the risk of recurrent violent injury by offering services to violence survivors before and following hospital discharge. These programs integrate case management, social service referrals, and community-based partnerships to support victims during their recovery and address underlying factors that contribute to violence [10]. Despite the increased prevalence of HVIPs emerging throughout the US in recent years and evidence of their potential benefits for reducing violent reinjury and retaliatory violence [9,11,12], rigorous evaluations of their effectiveness in reducing firearm-related harm are still needed, with a particular lack of adequately powered randomized controlled trials (RCTs) [8,13].

The Houston Hospital-Based Violence Intervention Program (Houston-HVIP) follows a two-phase research framework, beginning with an initial development and pilot phase to refine the intervention model (2023–2025), and followed by an anticipated RCT (2025–2028) to evaluate its impact on the primary outcome of firearm violence, as well as secondary outcomes of (a) violent reinjury, (b) attitudes toward violence, (c) post-traumatic stress, (d) aggression, and (e) self-rated health. This protocol paper provides an overview of the Houston-HVIP study design, objectives, methodology, and expected outcomes.

## Objectives and Aims

In 2022, the Community Firearm Violence Prevention (CFVP) Network was established. The CFVP Network comprises a Coordinating Center, a Steering Committee, and multiple research initiatives to evaluate community-based strategies to prevent firearm violence and associated injuries [14]. These initiatives foster collaboration between academic institutions and community organizations to develop, implement, and assess innovative, community-driven interventions to reduce firearm violence.

Research studies within the CFVP Network follow a structured two-phase research framework. The initial two-year developmental and pilot phase (UG3) focuses on developing, refining, and pilot testing the intervention. This is followed by a three-year (UH3) phase to evaluate the intervention’s effectiveness. Within the CFVP network, three projects were awarded funding from 2022–2027 (Cohort 1); three additional projects were funded from 2023–2028 (Cohort 2).

As part of Cohort 2, UTHealth Houston began the development of the Houston-HVIP in September 2023. The first two years (2023–2025) prioritized coalition-building by engaging internal and external stakeholders to guide program development and oversee its implementation. Additionally, this phase included a pilot study to test and refine the intervention. Upon completion of the pilot phase, a RCT with 274 GSW patients who present to the hospital with a GSW is planned to begin in September 2025. The UH3 phase has two aims:

**Aim 1** To determine the impact of the Houston-HVIP on the primary outcome of firearm violence.

**Aim 2** To determine the impact of the Houston-HVIP on the secondary outcomes of (a) violent reinjury, (b) attitudes toward violence, (c) post-traumatic stress, (d) aggression, and (e) self-rated health.

## Setting

Data collection for this study is scheduled for September 2025, with an 18-month recruitment period and a 12-month follow-up period. The study will occur at a large Level 1 trauma center within the Texas Medical Center—the world’s largest medical complex—in Houston, Texas [15]. The hospital is one of only two adult Level 1 trauma centers serving a population of over 7 million individuals. A review of hospital records found that 2,609 GSW patients were admitted between 2021 and 2024. In 2024, 685 persons presented to the hospital with a GSW, including 435 in our inclusion age range of 16–35. A distribution of GSW in 2024 by age is presented in Appendix A in S1 Appendix, which shows that 16–35 represents the highest prevalence of GSWs.

## Eligibility criteria

To be eligible for inclusion in the study, participants must meet the following criteria: (1) present to the hospital with a GSW, (2) be between 16 and 35 years of age, (3) speak English or Spanish, and (4) live within Harris County, Texas (the county which encompasses Houston). Exclusion criteria include: (1) GSW mechanism is (a) self-inflicted or (b) unintentional gunshot injury (i.e., being shot with a firearm without evidence of intent to harm), (2) being in police custody during the hospital admission, (3) primary residence is outside of Harris County, TX, and (4) being unable to provide informed consent due to inhibited psychological or development state.

## Eligibility screening

Program staff conducts eligibility screening by reviewing electronic medical records (EMRs) to identify patients presenting to the hospital with GSW. Eligibility screening occurs Monday through Friday (8:00 am – 5:00 pm) for GSW patients that presented to the hospital during the previous 24 hours. On Monday mornings, staff complete additional reviews for cases arriving from Friday evening (after 5:00 pm) to Monday at 8:00 am. The EMR review includes key patient details such as name, sex, race/ethnicity, mechanism of injury, hospital location (if admitted), and contact info if discharged. Following the initial EMR review, program staff consult with the patient care team—including nurses, physicians, residents, and advanced practice providers—to assess eligibility and the patient’s ability to provide informed consent, including factors such as pain levels, sedation status, and overall demeanor, which are critical to determining the patient’s readiness and willingness to learn about and consent to the program. If the medical team indicates that a patient is not yet in a condition to participate, program staff document the patient’s medical status using Research Electronic Data Capture (REDCap) [16,17] to schedule a follow-up assessment to be completed by program staff the next business day. This process repeats until a patient is deemed medically stable and able to provide consent and approached by a case manager to establish rapport and begin recruitment.

## Recruitment

**Fig 1:**
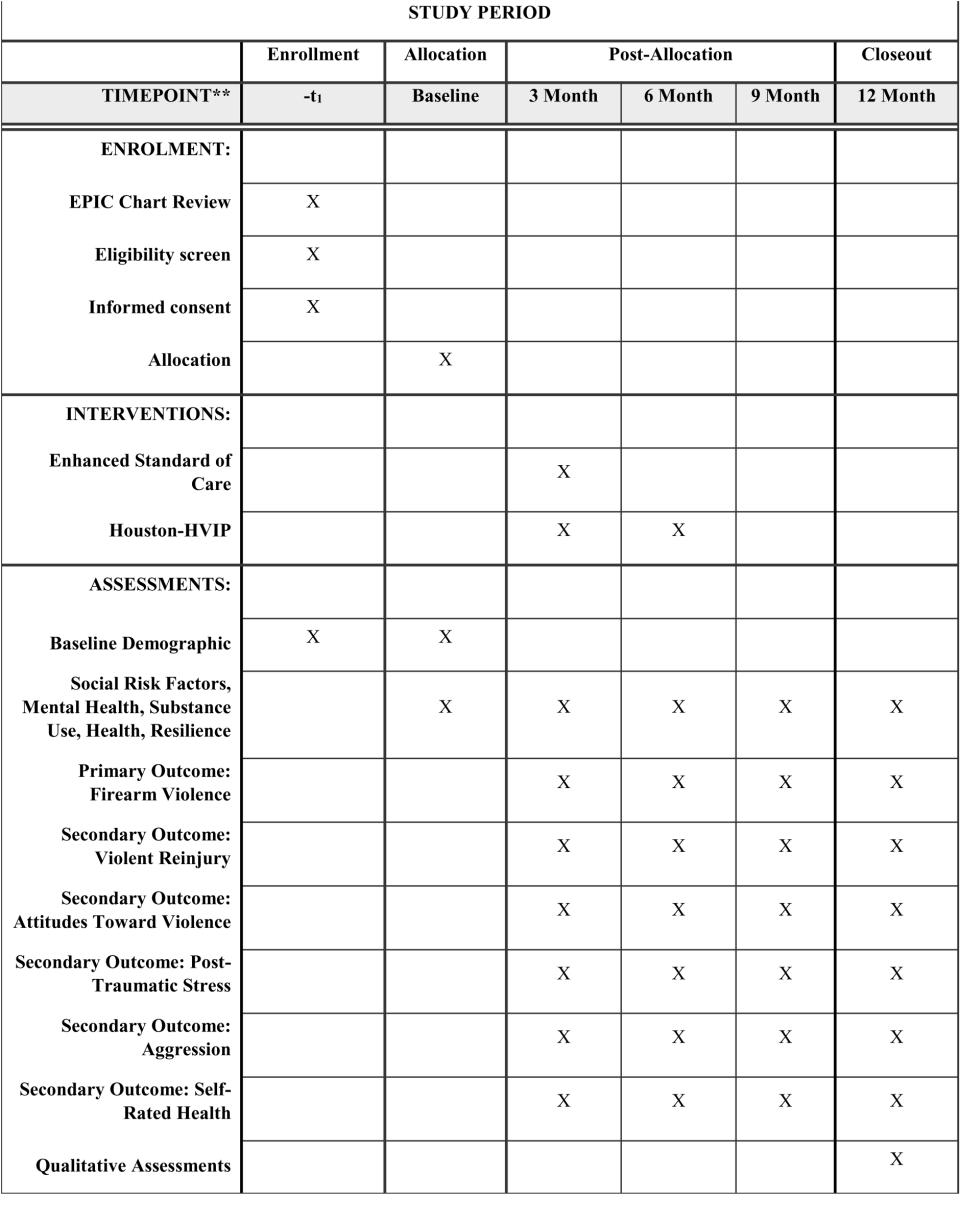
Schedule of enrollment, interventions, and assessments.

**Fig 2:**
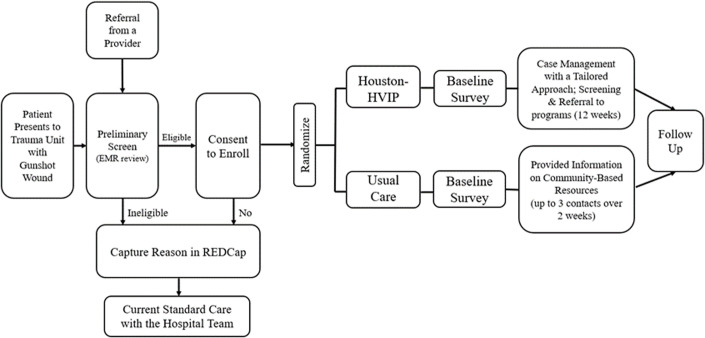
Study Recruitment Procedure.

Study recruitment procedures are detailed in Fig 1 and Fig 2. If a patient meets the initial eligibility criteria screening and no concerns are raised by medical staff regarding their ability to provide informed consent, a case manager will introduce the study at the bedside. Ideally, this introduction occurs during the initial interactions between the case manager and the patient. However, in some cases, multiple conversations may be necessary to establish rapport, build trust, and generate interest in the study. Case managers follow the rapport-building protocol outlined in Appendix B in S1 Appendix to facilitate this process.

To confirm eligibility, the case manager administers a four-item eligibility screener (see Appendix C in S1 Appendix). Once eligibility is confirmed, the patient will receive detailed information about the study and their right to decline participation. After reviewing and discussing the informed consent form, the case manager will obtain written consent from interested participants. Additionally, participants will complete a contact form for follow-up purposes, which includes (a) primary and secondary phone numbers, (b) email addresses, (c) home addresses, (d) secondary residential addresses, (e) emergency contact(s) information, and (f) social media usernames (if applicable).

For patients who meet eligibility criteria but are discharged before engaging with a case manager at the bedside, the case manager will make up to eight follow-up contact attempts over two weeks using the contact information in the EMR. Contact attempts are made via phone calls every other day. If phone calls are not answered, a voice mail is left, and an SMS text message is also sent (if the number listed is a cell phone). The case manager will attempt to reach individuals via telephone at various times and on different days of the week. Patients who decline participation at any time are no longer contacted. In addition, information on study materials and instructions on contacting the case manager are mailed to the participant at the address listed in the EMR. If a discharged patient expresses interest in participating after being contacted, arrangements will be made to meet them in person at the hospital or virtually via Microsoft Teams, Zoom, or a telephone call to discuss study details and obtain informed consent via an electronic consent process. A patient is eligible to enroll for up to one month following discharge.

Additionally, all trauma staff have been educated on the Houston-HVIP project and may share the study information with GSW patients. In these cases, medical staff have been trained and provided a badge with a QR code that links to information about the study and study staff contact information. If any hospital medical staff believes a patient is eligible for the program, a referral can be made by sending a patient’s information to Houston-HVIP study staff to begin the screening and consent process.

## Intervention/treatment

The RCT will be a parallel-group, two-arm trial with a 1:1 allocation ratio. After informed consent is obtained, participants are randomly assigned to either the treatment condition (Houston-HVIP) or the control condition (Standard of Care). Randomization is conducted in REDCap using permuted block randomization with varying block sizes. The randomization module in REDCap is pre-programmed with a computer-generated sequence to minimize selection bias and maintain allocation concealment. The targeted enrollment for the study is 274 participants (137 enrolled in each arm). The study will employ three full-time case managers, ensuring that each case manager is assigned no more than 10–12 high-intensity cases (i.e., cases in the treatment condition) at any given time during the six-month treatment period. Enrolled participants are randomly assigned to a case manager. This caseload aligns with the Health Alliance for Violence Intervention (HAVI) recommendations for the maximum recommended number of high-intensity cases per case manager and the appropriate duration of case management services for HVIPs [18]. Due to the nature of the planned intervention, participants and case managers will not be blinded to group assignment.

### Control condition: Enhanced usual care

The control condition is a “light-touch” intervention, where participants receive a one-off list of resources [19]. However, because of ethical concerns about identifying unmet social needs without offering support, participants will be provided with brief follow-up support from a case manager in the form of up to three contact attempts over two weeks to help answer questions about the provided information and address challenges navigating desired services. Accordingly, this goes beyond the typical standard care provided by the hospital system, and thus, we have conceptualized the control condition as “enhanced usual care.” Participants randomized to the control arm will receive the following:

1. **Indirect Resource Referral:** A list of community resources that address social needs (i.e., educational and financial needs and referrals for job training, educational support, housing assistance, and financial assistance programs).
2. **Trauma Survivors’ Network Engagement:** Information on opportunities for long-term engagement with the hospital’s trauma survivors’ network, which delivers peer support and various resources for survivors of traumatic injury, including twice-monthly virtual support groups and an online self-management/recovery course.
3. **Follow-Up Support:** A case manager will conduct up to three follow-up contacts over two weeks post-discharge to offer assistance with navigating community-based services. Contacts are made by a phone call; if a participant does not answer, both a text message and an email are sent.

### Treatment condition: Houston-HVIP program

The treatment condition is an intensive “high-touch” intervention where case managers provide ongoing assistance and direct referral to community-based organizations over six months [19]. The treatment condition is designed to meet the criteria for a high-intensity social needs intervention, which, based on a recent systematic review, includes (a) ≥8 contacts between a case manager and study participant, (b) meetings every 2 weeks, (c) planned meeting duration of ≥30 minutes, and (d) program duration of ≥6 months [20]. Participants in the Houston HVIP intervention arm will receive the following (see Table 1 for additional details on the week-by-week case management plan):

**Table 1:** Week-by-week case management plan.

Week	Case Management Activities
Week 1	Review participant screeners and identify immediate social needs Introduce available HVIP services Refer to violence interruption services if violence risk assessment score >3 Begin developing an individualized case management plan with the participant
Week 2	Present the individualized case management plan Identify and work to resolve barriers to accessing services Adjust case management plan to reflect current priorities
Week 4	Readminister social needs screener to identify new and ongoing social needs Identify and work to resolve barriers to accessing services Adjust case management plan to reflect current priorities
Week 6	Identify and work to resolve barriers to accessing services Adjust case management plan to reflect current priorities
Week 8	Readminister social needs screener to identify new and ongoing social needs Identify and work to resolve barriers to accessing services Adjust case management plan to reflect current priorities
Week 10	Identify and work to resolve barriers to accessing services Adjust case management plan to reflect current priorities
Week 12	Readminister social needs screener to identify new and ongoing social needs Identify and work to resolve barriers to accessing services Adjust case management plan to reflect current priorities Discuss with client long-term social needs and begin developing a long-term plan
Week 16	Readminister social needs screener to identify new and ongoing social needs Identify and work to resolve barriers to accessing services Adjust case management plan to reflect current priorities Review long-term plan and begin direct referrals to address long-term social needs
Week 20	Readminister social needs screener to identify new and ongoing social needs Identify and work to resolve barriers to accessing services Adjust case management plan to reflect immediate and long-term priorities
Week 24	Final check-in on referrals and outcomes Provide exit plan and long-term recommendations Administer participant satisfaction survey

***Note:*** Between weeks, the case manager makes direct referrals to community-based organizations on the participants’ behalf. A participant may contact a case manager at any point between weeks to request additional meetings as needed.

1. **Case Management and Risk Assessment:** A case manager will meet with each participant to assess social needs and risk factors for violent reinjury. This assessment will inform an individualized case management plan (see Appendices D and E in S1 Appendix).
2. **Personalized Social Service Linkages:** Case managers will work with a participant to identify their most immediate needs and use a direct referral model (i.e., the case manager forwards a patient’s contact information to a referral site contingent on the patient’s consent) to connect participants with tailored community-based services, addressing key social needs (i.e., job training, educational support, housing assistance, nutritional assistance, financial assistance) [21]. Every four weeks, the case manager will revisit the social service linkage plan and readdress social needs to ensure that the most immediate and pressing needs are consistently addressed. After three months, the case manager will also provide support in navigating to resources addressing the participants’ long-term social needs.
3. **Enhanced Violence Prevention Support:** Based on responses to the risk-assessment screener (see Appendix E in S1 Appendix), participants identified as being at elevated risk for violent reinjury will be referred to violence interrupter services.
4. **Trauma Survivors’ Network Engagement:** As with the control arm, participants will receive information about long-term engagement opportunities within the hospital’s trauma survivors’ network.

## Measurements

Upon enrollment, participants will complete a baseline survey administered in REDCap that collects information on demographics, socioeconomic status, behavioral health, and experiences with and attitudes toward violence. For this study, the survey instrument was designed in conjunction with the coordinating center and five other research programs via a harmonization data collection [22].

An overview of the survey domain included in the survey is provided in Appendix F in S1 Appendix. In addition to the baseline survey, participants will complete a social needs assessment and a violence risk assessment screener. The social needs assessment is used to identify social needs and to aid in the development of personalized case management plans for individuals enrolled in the Houston-HVIP (see Appendix E in S1 Appendix). The violence risk assessment, which is adapted from the Violent Reinjury Risk Assessment Instrument (VRRAI) [23], is used to evaluate participants’ likelihood of future violence involvement and informs case managers on which participants would benefit from referrals to violence interrupter programs (see Appendix E in S1 Appendix). The VRRAI, initially developed by the UCSF-Wraparound Program, is the only comprehensive risk assessment tool designed specifically for HVIPs.

To track changes over time, participants will also complete follow-up surveys every three months for one year (i.e., at 3-month, 6-month, 9-month, and 12-month follow-ups). These follow-up assessments include time-varying measures from the baseline survey, allowing for examining changes in key outcomes over the study period. The survey and all data collection instruments are offered in English and Spanish.

## Primary outcome

### Firearm violence

The primary outcome will be a composite event defined as the occurrence of a firearm violence event within the 12-month follow-up period, identified by any of three data sources to triangulate data and enhance event capture: (1) EMR records from all Level-1 trauma centers in Harris County, TX, will be evaluated to assess individuals who return to the hospital with a new GSW, (2) mortality records from the national death index will be searched to identify participants who died as a result of a GSW during the follow-up period, and (3) self-reported data from a survey instrument will be captured during each follow-up assessment to measure firearm violence using items from the Conflict Tactic Scales (CTS2) [24]. Before answering the CTS2 portion of the survey, respondents will receive a prompt asking them to “Think about some of the behaviors that happened to you during any fights, conflicts, arguments, or physical attacks with” (a) friends, neighbors, co-workers, or strangers, and (b) with a boyfriend/girlfriend, fiancé/fiancée, or husband/wife. Respondents are then asked, “In the past 3 MONTHS, including TODAY, how often did each of the following things happen:”

a. Friends, neighbors, co-workers, or strangers threatened me with a gun or used/fired a gun on ME: (a) never, (b) once, (c) twice, (d) 3–5 times, (e) 6–10 times, (f) 11–20 times, or (f) 20+ times.
b. MY PARTNER threatened me with a gun or used/fired a gun on ME: (a) never, (b) once, (c) twice, (d) 3–5 times, (e) 6–10 times, (f) 11–20 times, or (f) 20+ times.
c. I threatened them with a gun or used/fired a gun at THEM: (a) never, (b) once, (c) twice, (d) 3–5 times, (e) 6–10 times, (f) 11–20 times, or (f) 20+ times.
d. I threatened them with a gun or used/fired a gun at MY PARTNER: (a) never, (b) once, (c) twice, (d) 3–5 times, (e) 6–10 times, (f) 11–20 times, or (f) 20+ times.

## Secondary outcomes

### Firearm and non-firearm victimization

Firearm and non-firearm victimization will be measured using the same criteria as the primary outcome, but will focus on information on cases pertaining to (a) hospital readmission for both firearm and non-firearm violent injury stemming from interpersonal violence (i.e., stabbings; assaults), (b) death due to a firearm or non-firearm violent injury, and (c) self-report for violence from firearms as well as non-firearms. Items from the CTS-2 will use the same prompts above and will include the following additional questions.

a. MY PARTNER pushed, shoved, or slapped ME: (a) never, (b) once, (c) twice, (d) 3–5 times, (e) 6–10 times, (f) 11–20 times, or (f) 20+ times.
b. MY PARTNER kicked, punched, beat me up, or threatened/used a knife or something sharp on ME: (a) never, (b) once, (c) twice, (d) 3–5 times, (e) 6–10 times, (f) 11–20 times, or (f) 20+ times.
c. THEY pushed, shoved, or slapped ME: (a) never, (b) once, (c) twice, (d) 3–5 times, (e) 6–10 times, (f) 11–20 times, or (f) 20+ times.
d. THEY kicked, punched, beat me up, or threatened/used a knife or something sharp on ME: (a) never, (b) once, (c) twice, (d) 3–5 times, (e) 6–10 times, (f) 11–20 times, or (f) 20+ times.

### Attitudes toward firearm violence

Attitudes toward violence are measured via self-reported survey items listed below, all of which include response options (strongly agree, agree, disagree, strongly disagree) [25]:

a. It is okay to shoot a person if that is what it takes to get something you want.
b. It is okay to use a gun to shoot someone who insults or hurts you (for example: beats you up).
c. Carrying a gun makes you feel powerful/potent.
d. Carrying a gun makes you safer.
e. It’s okay to carry a gun if you live in a rough neighborhood.
f. It is okay to use a gun to threaten someone who disrespects you.

### Post-Traumatic Stress Disorder Symptoms (PTSD)

Symptoms of PTSD are measured using items from the Post-Traumatic Stress Disorder Checklist Version 2 (PCL-2) [26]. Specifically, respondents were asked in the past month how much they were bothered by (responses not at all, a little bit, quite a bit, extremely):

a. Repeated, disturbing, and unwanted memories of the stressful experience?
b. Feeling very upset when something reminded you of a stressful experience?

### Aggression

Aggression is measured using self-reported items from the Copeland-Linder Scale [27] (response options: strongly agree, agree, disagree, strongly disagree):

a. I believe that if someone hits you, you should hit them back.
b. I believe revenge is a good thing.
c. I believe that it is OK to hurt people if they hurt you first.

### Self-rated health

Self-rated health is measured using a single survey item asking respondents “In general, would you say your health is?” (excellent, very good, good, fair, poor)

## Participant timeline

In addition to the baseline assessment, study participants will provide follow-up data at four additional time points: 3 months, 6 months, 9 months, and 12 months post-enrollment (see Fig 1 for details of the participant timeline). Surveys are administered electronically through REDCap. Survey compensation is $50 for the baseline survey and $100 for follow up surveys. Following the final 12-month survey, participants will also be asked to complete a program satisfaction survey (see Appendix G in S1 Appendix) [28]. Additionally, a subset of participants (*n* = 40) will be invited to participate in semi-structured follow-up interviews, each lasting approximately 60 minutes, to gain deeper insights into their experiences with the intervention [29]. Participants will be compensated $100 for the follow-up interview.

## Sample size/Statistical power

The sample size calculation is based on a comparison of the primary outcome of firearm violence between two randomization groups (Enhanced Usual Care vs. Houston-HVIP). We will extract the event dates for events captured in the first two data sources (EMR and mortality records), while the CTS2 measured at 3, 6, 9, and 12 months following enrollment will capture whether the event occurs between the visits. The first occurrence captured in the three data sources will then be determined for the composite event., We will compare the occurrence of firearm violence between the two arms using a Weibull regression model for the interval- and right-censored data to account for the interval-censoring (due to the planned measurement schedule for CTS2) and right-censoring (due to potential loss to follow-up and administrative censoring at 12 months). A prior study among youth seeking assault-injury care at an emergency department observed a two-year rate of 59% for a similarly-defined firearm violence composite, where the reporting rates between 0–6, 6–12, 12–18, and 18–24 months were 37.5%, 25.8%, 20.5%, and 15.2% respectively [30]. This pattern suggests a potential decreasing hazard over time that can be well accounted for by a Weibull distribution, which flexibly allows for increasing, decreasing, or constant hazard shapes. The null hypothesis of no intervention effect corresponds to a regression coefficient of zero for the study group variable or equivalently a hazard ratio (HR) of 1.

We conducted statistical simulations based on a Weibull distribution with shape and scale parameters chosen according to rates in the prior study, such that the twelve-month probability is 42% in the SOC arm. To estimate the power, we generated 10,000 simulated data from this distribution, imposed interval censoring according to the planned follow-up, added right censoring from potential attrition (patient lost to follow-up), and applied the Weibull regression model with a two-tailed Wald test. Under a sample size of 274 (137 in each arm) and allowing up to 35% attrition during the follow-up, we have 80% power to detect a HR of 0.50 with a two-sided alpha of 0.05, corresponding to a reduction from 42% to 24% in twelve-month probability. The planned adjustment of important risk factors in the Weibull regression model will offer enhanced power. The planned sample size will also provide adequate power for the secondary outcomes. For example, for a continuous outcome, a complete sample size of 200 (100 + 100) allows us to detect a small to moderate between-group difference, Cohen’s d = 0.40, with 80% power and a two-sided alpha = 0.05 in a two-sample t-test.

## Consent

All participants will provide written informed consent and authorizations before randomization, in accordance with UTHealth institutional review board requirements. For individuals under 18, parental/legal guardian consent will be required, along with the minor’s assent, prior to randomization.

## Data management

Data for this study will be collected using REDCap, with access restricted to the data manager, program manager, and case managers, all of whom will remain unblinded to study conditions. This limited access is intentional to minimize data entry errors, reduce the number of individuals responsible for data management, and maintain the integrity of the study by ensuring that personnel blinded to randomization conditions cannot access raw data files. To protect participant confidentiality, all data within the REDCap database will be de-identified.

## Statistical methods

The primary analysis will follow the intention-to-treat principle. A Weibull regression model for interval- and right-censored data will be used to compare the occurrence of firearm violence between the two study groups. A two-sided alpha of 0.05 will be used to determine statistical significance. Since prespecified adjustment of important baseline covariates can provide enhanced power, the regression model will be adjusted for race/ethnicity, biological sex, and age. In addition, we plan to balance-cross case managers across the two arms to protect against potential agent effects, if any, and will adjust the case manager as a fixed factor covariate in our analytic model [31].

Next, to assess the potential heterogeneity of the intervention effect, we will examine interactions between the randomization group and important baseline risk factors (e.g., race/ethnicity) in additional secondary analyses. The corresponding interaction terms will be added individually to the regression model to examine potential effect modification. We will also carry out a per-protocol analysis as a sensitivity analysis.

The secondary outcomes that are measured longitudinally (e.g., PSTD) will be analyzed using appropriate longitudinal models, such as the (generalized) linear mixed model with a suitable distribution and link function, including group, time (categorical), group-by-time interaction, as well as subject-specific random effects to account for the repeated measures. We will first conduct a contrast to test the intervention effect across the post-baseline time points. Group-specific means and between-group differences will also be estimated at each time point with 95% confidence intervals.

## Ethical considerations

All study procedures have been approved by the UTHealth Houston Committee for the Protection of Human Subjects (HSC-MS-23–0904) and the NIH. The study is registered on ClinicalTrials.gov (ID: NCT06263647). The study is overseen by a data monitoring and safety board at UTHealth Houston. Any adverse or serious adverse events will be reported to the study principal investigators, data monitoring and safety board, and institutional review board within 24 hours.

## Contingency plans

In the event that participant recruitment falls short of the target enrollment of 274 individuals, we have developed a set of proposed contingency plans in consultation with the University of Michigan Coordinating Center and the Houston-HVIP Community Advisory Board. These plans are precautionary in nature and do not represent proposed changes to the UH3 phase at this time. Implementation of any contingency strategies will occur only with NIH approval and only if recruitment challenges arise under the current eligibility criteria.

1. **Expanding the Eligible Age Range:** We may consider increasing the upper age limit for participant eligibility from 35 to 40 years. Hospital data from 2024 suggest that this expansion would add approximately 89 additional eligible participants over an 18-month recruitment period.
2. **Broadening the Geographic Catchment Area:** Current eligibility is limited to residents of Harris County. TX. If necessary, we may expand eligibility to include individuals residing within a 40-mile radius of MHH–TMC, encompassing surrounding counties and municipalities. Based on 2024 data, this modification could add approximately 53 additional eligible participants aged 16–35.
3. **Extending the Recruitment Window:** The original study design specifies an 18-month recruitment period. If enrollment lags, we are prepared to extend this period up to 24 months. This adjustment would reduce the required average monthly enrollment from approximately 15–11 participants.
4. **Expanding Eligible Injury Types:** The current inclusion criteria are limited to individuals presenting with GSWs. If needed, we may expand eligibility to include patients with other penetrating violent injuries, such as stab wounds. Hospital data from 2024 indicate that this expansion would add approximately 105 eligible participants over an 18-month period.

## Discussion

### Strengths

This study design addresses several limitations identified in prior reviews of HVIPs.(13) First, by implementing a two-arm RCT, this study may contribute to the HVIP literature by evaluating the effectiveness of intensive case management services compared to a control condition of usual care with a causal inference design. Second, many HVIP studies are limited by small sample sizes and insufficient statistical power, making it difficult to assess program effectiveness. In contrast, this study aims to recruit approximately 274 participants, providing adequate statistical power and positioning it as one of the largest HVIP RCTs conducted to date [13]. Third, while most HVIP evaluations focus on reducing violent reinjury, few examine broader outcomes such as attitudes toward aggression, attitudes toward violence, as well as mental and overall health. Fourth, we employ a one-year follow-up period with data collection at 3, 6, 9, and 12 months, extending beyond the six-month post-injury follow-up period that is common in many prior HVIP studies [32]. Fifth, our approach to measuring violent reinjury is multifaceted, leveraging (a) hospital readmission data covering both adult Level 1 trauma centers in the Greater Houston area, (b) comprehensive national death record searches, and (c) self-reported information using a validated instrument. Sixth, the intervention arm has also been designed to be client-centered to ensure that patients’ reported needs are considered and consistently updated so that social needs service referrals and connections address those underlying needs [33]. Finally, responding to calls from scholars, this study includes a qualitative assessment to examine the impact of the HVIP program on participant experiences and outcomes [29].

### Limitations

Despite the strengths of this study, there are limitations of the current study that can potentially be addressed by future HVIPs. First, the study focuses on GSW patients, limiting the generalizability of findings to individuals with other types of violent injuries, such as stabbings or assaults. Second, enrollment is restricted to individuals aged 16–35, a range selected based on hospital EMR data indicating the highest incidence of gunshot wounds within this age group. The decision to enroll minors aged 16–17 was primarily because they are treated in the adult trauma unit while enrolling patients under 16 would require coordination with the affiliated children’s hospital trauma unit. Consequently, the findings may not be applicable to GSW victims outside of the targeted age range. Third, study participation is limited to individuals who are fluent in English or Spanish. While this criterion encompasses the vast majority of GSW patients presenting to the hospital, it restricts the ability to assess HVIP effectiveness among those who speak other languages. Additionally, the study excludes individuals with self-inflicted or unintentional gunshot wounds, preventing conclusions about the role of HVIPs in reducing the recurrence of these types of injuries. Finally, the study is conducted at a large, urban, Level 1 trauma center, which provides a high volume of GSW patients for recruitment. However, this setting may limit the generalizability of findings to other geographic regions or hospital types.

### Dissemination

Findings will be disseminated through peer-reviewed journal publications, meetings with local hospital administrators, and presentations at local, regional, and national conferences.

## Conclusion

Firearm violence imposes substantial social and economic burdens on society. In response, HVIPs have been increasingly implemented in cities across the US to support victims of violence by addressing their needs before discharge. However, despite the growing body of research on HVIPs, their effectiveness remains inconsistent, and there is a lack of large-scale RCTs assessing their impact on violent reinjury [13]. To fill this gap, we anticipate conducting an RCT at a major Level 1 trauma center in the fourth-largest city in the United States. Findings from this study will provide critical insights to inform the design and implementation of future HVIP programs.

## Supporting information

S1 Appendix (DOCX)

S1 File (PDF)

S1 Protocol (DOCX)

## References

[1] CDC Wonder. About Underlying Cause of Death, 2018-2022, Single Race. 2024. https://wonder.cdc.gov/controller/saved/D158/D362F919 2024 September 26

[2] Cook A, Osler T, Hosmer D, Glance L, Rogers F, Gross B, et al. Gunshot wounds resulting in hospitalization in the United States: 2004-2013. Injury. 2017 Mar;48(3):621–7. 28173921 10.1016/j.injury.2017.01.044

[3] Kaufman EJ, Song J, Xiong R, Seamon MJ, Delgado MK. Fatal and Nonfatal Firearm Injury Rates by Race and Ethnicity in the United States, 2019 to 2020. Ann Intern Med. 2024;177(9):1157–69. doi: 10.7326/M23-2251 39074371

[4] Vella MA, Warshauer A, Tortorello G, Fernandez-Moure J, Giacolone J, Chen B, et al. Long-term Functional, Psychological, Emotional, and Social Outcomes in Survivors of Firearm Injuries. JAMA Surg. 2020;155(1):51–9. doi: 10.1001/jamasurg.2019.4533 31746949 PMC6902182

[5] Miller GF, Barnett SBL, Florence CS, McDavid Harrison K, Dahlberg LL, Mercy JA. Costs of Fatal and Nonfatal Firearm Injuries in the U.S., 2019 and 2020. Am J Prev Med. 2024;66(2):195–204. doi: 10.1016/j.amepre.2023.09.026 38010238 PMC10843794

[6] Miller T, Downing J, Wheeler L, Fischer K. The Medical Costs of Firearm Injuries in the United States: A Systematic Review. J Emerg Med. 2024;66(2):109–32. doi: 10.1016/j.jemermed.2023.08.013 38262782

[7] Affinati S, Patton D, Hansen L, Ranney M, Christmas AB, Violano P, et al. Hospital-based violence intervention programs targeting adult populations: an Eastern Association for the Surgery of Trauma evidence-based review. Trauma Surg Acute Care Open. 2016;1(1):e000024. doi: 10.1136/tsaco-2016-000024 29766064 PMC5891700

[8] Strong BL, Shipper AG, Downton KD, Lane WG. The effects of health care-based violence intervention programs on injury recidivism and costs: A systematic review. J Trauma Acute Care Surg. 2016;81(5):961–70. doi: 10.1097/TA.0000000000001222 27537505

[9] Bonne S, Dicker RA. Hospital-based violence intervention programs to address social determinants of health and violence. Curr Trauma Rep. 2020;6(1):23–8.

[10] The Health Alliance For Violence Intervention. Hospital-based violence intervention programs (HVIPs). The HAVI. https://www.thehavi.org/what-is-an-hvip. 2025 March 12

[11] Nofi CP, Roberts BK, Cornell E, Tijerina M, Tussing O, Henry MC. Hospital-based violence intervention programs to reduce firearm injuries in children: a scoping review. J Pediatr Surg. 2023;58(11):2212–21. 37217364 10.1016/j.jpedsurg.2023.04.020

[12] Affinati S, Patton D, Hansen L, Ranney M, Christmas AB, Violano P, et al. Hospital-based violence intervention programs targeting adult populations: an Eastern Association for the Surgery of Trauma evidence-based review. Trauma Surg Acute Care Open. 2016;1(1):e000024. doi: 10.1136/tsaco-2016-000024 29766064 PMC5891700

[13] Webster DW, Richardson J, Meyerson N, Vil C, Topazian R. Research on the effects of hospital-based violence intervention programs: Observations and recommendations. Ann Am Acad Pol Soc Sci. 2022;704(1):137–57.

[14] The Community Firearm Violence Prevention Network. https://endfirearmviolence.org/. 2025 March 12

[15] United States Census Bureau. Census.gov. Census Population Clock. 2024 2025 Mar 12 https://www.census.gov/popclock/embed.php?component=populous

[16] Harris PA, Taylor R, Thielke R, Payne J, Gonzalez N, Conde JG. Research electronic data capture (REDCap)—A metadata-driven methodology and workflow process for providing translational research informatics support. J Biomed Inform. 2009;42(2). 10.1016/j.jbi.2008.08.010 PMC2700030 18929686

[17] Harris PA, Taylor R, Minor BL, Elliott V, Fernandez M, O’Neal L, et al. The REDCap consortium: Building an international community of software platform partners. J Biomed Inform. 2019;95:103208. 31078660 10.1016/j.jbi.2019.103208 PMC7254481

[18] The Health Alliance For Violence Intervention. Standards and indicators for hospital-based violence intervention programs (HVIPs). Boston, MA: The Health Alliance For Violence Intervention. 2022. https://www.google.com/

[19] Neadley KE, Shoubridge C, Lynch JW, Boyd MA, Poirier BF. Hospital-Based Interventions Addressing Social Needs: A Systematic Narrative Review. Am J Prev Med. 2025;:107624. doi: 10.1016/j.amepre.2025.03.004 40154814

[20] Viswanathan M, Kennedy SM, Sathe N, Eder ML, Ng V, Kugley S, et al. Evaluating Intensity, Complexity, and Potential for Causal Inference in Social Needs Interventions. JAMA Netw Open. 2024;7(6):e2417994. 10.1001/jamanetworkopen.2024.17994 PMC11193129 38904959

[21] Ruiz Escobar E, Pathak S, Blanchard CM. Screening and referral care delivery services and unmet health-related social needs: a systematic review. Prev Chronic Dis. 2021;18:E78. 10.5888/pcd18.200569 PMC8388203 34387188

[22] Sokol R, Walton M, Lee D, Seewald L, Del Toro VM, Farooqui M, et al. Advancing Science to Prevent Firearm Violence in Communities: A Process for Harmonizing Studies to Develop Research Infrastructure. Prev Sci. 2024;25(7):1122–32. doi: 10.1007/s11121-024-01723-5 39304578 PMC13051374

[23] Kramer EJ, Dodington J, Hunt A, Henderson T, Nwabuo A, Dicker R, et al. Violent reinjury risk assessment instrument (VRRAI) for hospital-based violence intervention programs. J Surg Res. 2017;217:177-186.e2. doi: 10.1016/j.jss.2017.05.023 28602221 PMC5647140

[24] Straus MA, Hamby SL, Boney-McCoy S, Sugarman DB. The revised conflict tactics scales (CTS2): Development and preliminary psychometric data. J Fam Issues. 1996;17(3):283–316.

[25] Shapiro JP, Dorman RL, Burkes WM, Welker CJ, Clough JB. Development and factor analysis of a measure of youth attitudes toward guns and violence. J Clin Child Psychol. 1997;26(3):311–20. 9292389 10.1207/s15374424jccp2603_10

[26] Lang AJ, Stein MB. An abbreviated PTSD checklist for use as a screening instrument in primary care. Behav Res Ther. 2005;43(5):585–94. 15865914 10.1016/j.brat.2004.04.005

[27] Copeland-Linder N, Jones VC, Haynie DL, Simons-Morton BG, Wright JL, Cheng TL. Factors associated with retaliatory attitudes among African American adolescents who have been assaulted. J Pediatr Psychol. 2007;32(7):760–70. 17403911 10.1093/jpepsy/jsm007

[28] Myers RK, Kapa HM, Garcia SM, Vega L, Fein JA. Development of a brief client satisfaction and quality improvement tool for hospital-based violence intervention programs: opportunities for enhancing client perspectives. J Patient Exp. 2025;12:23743735251314622. doi: 10.1177/23743735251314622 39830314 PMC11742158

[29] Ranjan S, Neudecker CH, Strange CC, Wojcik MLT, Shah A, Solhkhah R. Hospital-Based Violence Intervention Programs (HVIPs): Making a Case for Qualitative Evaluation Designs. Crime Delinquency. 2023;69(3):487–509.

[30] Carter PM, Walton MA, Roehler DR, Goldstick J, Zimmerman MA, Blow FC, et al. Firearm violence among high-risk emergency department youth after an assault injury. Pediatrics. 2015;135(5):805–15. doi: 10.1542/peds.2014-3572 25847808 PMC4411782

[31] Moyer JC, Li F, Cook AJ, Heagerty PJ, Pals SL, Turner EL. Evaluating analytic models for individually randomized group treatment trials with complex clustering in nested and crossed designs. Statistics in Medicine. 2024;43(25). 10.1002/sim.10206 39225281

[32] St Vil C, Hall EC, Sheppard M, Williams M. Testing the efficacy of a hospital-based violence intervention programme: protocol and design. Inj Prev. 2020. doi: injuryprev–2020–044026 10.1136/injuryprev-2020-044026 33328172

[33] Jang A, Thomas A, Slocum J, Tesorero K, Danna G, Saklecha A, et al. The gap between hospital-based violence intervention services and client needs: A systematic review. Surgery. 2023;174(4):1008–20. doi: 10.1016/j.surg.2023.07.011 37586893

