# Supplementary material for: Protocol for the Houston Hospital-based violence intervention program
Source: PLoS One. 2025 Jul 2;20(7):e0325569. doi: 10.1371/journal.pone.0325569 (PMC12221051; doi:10.1371/journal.pone.0325569)
Supplement: S1 Appendix — (DOCX) [file pone.0325569.s001.docx]

**Appendix A: Distribution of GSW Patients by Age in 2024 (*N* = 685)**


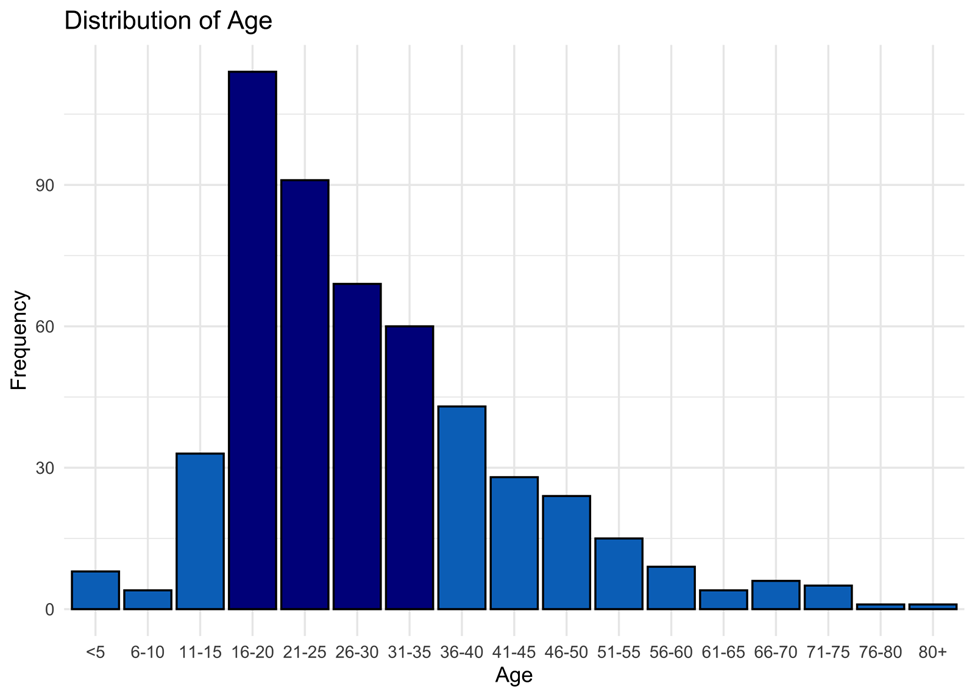
*Note:* 435 individuals in the inclusion age range of 16-35 presented for a GSW in 2024.

**Appendix B: Rapport Building Script**

*This is a* ***GUIDE*** *that the case manager can use during the first interaction with the patient when the patient is medically stable. Recruitment and retention success highly depends on the initial contact and the relationship we establish with the patient/participant. It is important that case managers and study team members build trust with the patients/participants by making eye contact when speaking with them and letting them know that you’re listening to them. It is important to connect with the patient by asking questions and support them by responding to their needs and requests.*

Introduction

Hi my name is (*case manager’s name*), and I am a case manager at UTHealth. Is it OK if I call you (check with clinical staff/EMR))? I am working on a program to help individuals who have experienced gun injuries and will try to connect you to resources to help you. I’m also here to offer support and gather some information that will help us improve our services for patients in similar positions.

Building rapport:

*Express Empathy:* " How are you feeling today, both physically and emotionally?"

*Show Genuine Concern:* "It's important to us that you feel supported during your recovery. Is there anything specific you need right now or any immediate concerns you’d like to talk about?"
Is there anyone you’d like us to contact right now?

If you don’t mind my asking, can you tell me about the events that led to your hospital visit?

*Listen Actively:* "How do you feel like you are/were treated when you were admitted to the hospital?

*Personal Connection:* “Do you have a place to stay right now? What is your work situation? If you’re comfortable, could you share a little about yourself, such as your interests or what you enjoy doing?"

Interaction with Medical Staff:
*Build Trust:* "How would you describe your experience with the medical staff? Were they understanding and professional in regard to your needs/care?"

*Ensure Clarity:* "Were you given clear and understandable information about your diagnosis and treatment plan?"

Concerns and Needs:
*Acknowledge Their Concerns:* "Were there any specific concerns or difficulties you encountered during your hospital visit that we haven’t discussed that you would like me to address? How can we address them better in the future?"

*Offer Reassurance:* "Our goal is to ensure you feel heard and supported. Did you feel that your needs were met during your time in the hospital?"

After the patient completes the survey/before the case manager leaves the participant:

"Thank you so much for taking the time to share your experiences with me. Your feedback is crucial in helping us improve our services and support for other gunshot wound patients. If you have any further questions or need additional support, please don't hesitate to reach out. I hope you have a speedy recovery.”

**Appendix C: Eligibility Screening Form**

**Study ID: ____________**

**Screening date: __ __ / __ __ / __ __ __ __**

**MM DD YYYY**

**Visit:** Screening

**Completed by: Study personnel via EHR or in person**

| **No** | **Yes** | **Inclusion Criteria** | | |
| --- | --- | --- | --- | --- |
| □ | □ |  | | Between ages 16-35 years old (at time of enrollment)  □ Verified by EPIC □ Verified with participant/parent □ Verified with provider  NOTES: |
| □ | □ |  | | Has a gunshot injury  □ Verified by EPIC □ Verified with participant/parent □ Verified with provider  NOTES: |
| □ | □ |  | | Primary language is English or Spanish  □ Verified by EPIC □ Verified with participant/parent □ Verified with provider NOTES: |
| □ | □ |  | | Primary residence is in Harris County (at time of enrollment) □ Verified by EPIC □ Verified with participant/parent  □ Verified with provider NOTES: |
|  |  |  | |  |
| **No** | **Yes** | **Exclusion Criteria** | | |
| □ | □ |  | Source of firearm injury is self-inflicted or unintentional  □ Verified by EPIC □ Verified with participant/parent □ Verified with provider  NOTES: | |
| □ | □ |  | Actively detained by criminal justice systems (at time of enrollment)  NOTES:  □ Verified by EPIC □ Verified with participant/parent □ Verified with provider | |
| □ | □ |  | Deceased upon or after admission  NOTES:  □ Verified by EPIC □ Verified with participant/parent  □ Verified with provider | |

NOTES: (Additional notes)

**Appendix D: Social Risk Screening**

Please tell me if you are currently concerned about any of the following for yourself. The answers are YES/No.

| 1. Running out of food before having enough money or food stamps to buy more. | If YES, would you like help with this? | ☐ Yes ☐ No ☐ Maybe |
| --- | --- | --- |
| 1. Unstable housing, including eviction, foreclosure, homelessness, or staying with friends/family. | If YES, would you like help with this? | ☐ Yes ☐ No ☐ Maybe |
| 1. Problems paying bills, like electric, gas, water, or phone bills | If YES, would you like help with this? | ☐ Yes ☐ No ☐ Maybe |
| 1. Housing problems like mold, insects, rats, or mice | If YES, would you like help with this? | ☐ Yes ☐ No ☐ Maybe |
| 1. Difficulty finding a job | If YES, would you like help with this? | ☐ Yes ☐ No ☐ Maybe |
| 1. Do you interested in starting or continuing school or job training? or example, working toward a high school diploma, GED, or completing a training program | If YES, would you like help with this? | ☐ Yes ☐ No ☐ Maybe |
| 1. Difficulty quitting or cutting down on using too much alcohol or drugs | If YES, would you like help with this? | ☐ Yes ☐ No ☐ Maybe |
| 1. A disability interfering with the ability to work | If YES, would you like help with this? | ☐ Yes ☐ No ☐ Maybe |
| 1. Problems with a current or former job, like unpaid wages, workers comp, discrimination or harassment | If YES, would you like help with this? | ☐ Yes ☐ No ☐ Maybe |
| 1. Difficulty obtaining unemployment insurance | If YES, would you like help with this? | ☐ Yes ☐ No ☐ Maybe |
| 1. Getting cut off from or denied from programs that provide income support, like the Supplemental Nutrition Assistance Program (food stamps) or unemployment benefits. | If YES, would you like help with this? | ☐ Yes ☐ No ☐ Maybe |
| 1. Having no health insurance | If YES, would you like help with this? | ☐ Yes ☐ No ☐ Maybe |
| 1. Having no primary care provider | If YES, would you like help with this? | ☐ Yes ☐ No ☐ Maybe |
| 1. Receiving medical or pharmacy bills that you cannot afford | If YES, would you like help with this? | ☐ Yes ☐ No ☐ Maybe |
| 1. Difficulty finding childcare | If YES, would you like help with this? | ☐ Yes ☐ No ☐ Maybe |
| 1. Concerns about your mental health | If YES, would you like help with this? | ☐ Yes ☐ No ☐ Maybe |
| 1. Difficulty with transportation | If YES, would you like help with this? | ☐ Yes ☐ No ☐ Maybe |
| 1. Concerns about your safety | If YES, would you like help with this? | ☐ Yes ☐ No ☐ Maybe |

*Note*: The screening tool is adapted from the following studies:

Garg, A., Toy, S., Tripodis, Y., Silverstein, M., & Freeman, E. (2015). Addressing social determinants of health at well childcare visits: a cluster RCT. *Pediatrics*, *135*(2), e296-e304.

Gottlieb, L. M., Adler, N. E., Wing, H., Velazquez, D., Keeton, V., Romero, A., Hernandez, M., Vera, A. M., Caceres, E. U., Arevalo, C., Herrera, P., Suarez, M. B., & Hessler, D. (2020). Effects of in-person assistance vs personalized written resources about social services on household social risks and child and caregiver health: a randomized clinical trial. *JAMA Network Open*, *3*(3), e200701-e20070

**Appendix E: Risk Assessment**

1. Do you currently feel that you or someone close to you is in immediate danger of being harmed due to an ongoing conflict?

☐ Yes

☐ No

1. Are you a member of a gang or do you regularly spend time with individuals who are associated with a gang?

☐ Yes

☐ No

1. Including the event that led you to the hospital how many times have you previously been (a) shot, or (b) stabbed?
   1. Number of shootings: ____________
   2. Number of Stabbings: ____________

**Personal incarceration
*Family History of Incarceration Study (FamHIS) - Release***

1. Have you ever spent time in a jail, prison, juvenile detention center or other correctional facility?

☐ Yes

☐ No

- 1. Was your incarceration related to a violent crime (e.g., assault, robbery, or other offenses involving physical harm or the threat of harm)?

☐ Yes

☐ No

These next questions ask about people you may have known who were killed by a gun. We know these are personal questions and all your answers are private.

If you are experiencing any distress related to this question, please call either the National Crisis Hotline at 988 or notify the Research Assistant (RA) and we will provide helpful resources.

1. How many of your friends or family members (for example, your Mom, Dad, Sister/Brother, Cousin) have ever been shot or killed with a gun?

☐ 0

☐ ….

☐ 25+

*Note:* Screening tool is adapted from: Kramer, E. J., Dodington, J., Hunt, A., Henderson, T., Nwabuo, A., Dicker, R., & Juillard, C. (2017). Violent reinjury risk assessment instrument (VRRAI) for hospital-based violence intervention programs. *Journal of Surgical Research*, *217*, 177-186.

**Appendix F: Houston-HVIP Baseline Survey Domains**

|  |  |  |
| --- | --- | --- |
| **Demographic Characteristics:**  Age  Education School  Employment Status  Employment Hours  Employment Type  Household Income  Household size  Children in the home Race Sex Sexual orientation Relationship status | **Non-firearm Violence:**  Community violence exposure  Partner and Non-partner victimization  Partner and Non-partner aggression  Physical Fighting  Attitudes toward aggression and violence  Criminal justice consequences  Avoidance behaviors  Violence prevention self-efficacy | **Firearms and firearm violence:**  Firearm violence exposure  Firearm violence attitudes  Firearm ownership  Firearm carriage  Firearm carriage reasons  Risky firearm behaviors  Risky Firearm Discharge Behaviors  SaFETy score |
| **Social Risk Factors** | **Adverse Childhood Experiences** |  |
| **Personal incarceration** | **Social media** |  |
| **Mental Health:**  Depression  Anxiety  Loneliness PTSD | **Risk Assessment** |  |
| **Substance Use:**  Alcohol use  Drug use |  |  |
| **Physical Health:**  General Health  Sleep medication Daytime dysfunction |  |  |
| **Resilience** |  |  |

**Appendix G: Patient Satisfaction Survey**

| **Item** | **Response Options** |
| --- | --- |
| Using any number from 0 to 10, what number would you use to rate the services you received from (case manager name) while in the program? | 0 (Worst)–10 (Best) |
| To what extent has our program met your needs? | All my needs were met, Most of my needs were met, A few of my needs were met, None of my needs were met |
| How happy are you with the amount of help you received? | Very happy, Mostly happy, Neither happy nor unhappy, Quite unhappy |
| Did the program help you to feel better? | It helped me a great deal, It helped somewhat, It did not help, It made me feel worse |
| Overall, how happy were you with the services you received? | Very happy, Mostly happy, Neither happy nor unhappy, Quite unhappy |
| If you were to experience violence again, would you choose to come back to our program? | Definitely, Maybe, Probably not, Definitely not |
| How often did the program staff listen carefully to you? | Always, Usually, Sometimes, Never |
| Program staff respect parts of your culture or identity that are important to you. | Always, Usually, Sometimes, Never |
| Because of the program, you know more about community resources that are available to you. | Definitely, Maybe, Probably not, Definitely not |
| Because of the program, you feel better prepared to access community resources, such as legal, medical, educational, or mental health services. | Definitely, Maybe, Probably not, Definitely not |
| You are prepared to apply the skills and tools you learned during the program. | Definitely, Maybe, Probably not, Definitely not |
| Is there anything else you would like to share about (program name), such as what was helpful or unhelpful or ways that (program name) could be improved? | Free text |

Note: Survey adapted from: Myers, R. K., Kapa, H. M., Garcia, S. M., Vega, L., & Fein, J. A. (2025). Development of a Brief Client Satisfaction and Quality Improvement Tool for Hospital-Based Violence Intervention Programs: Opportunities for Enhancing Client Perspectives. *Journal of Patient Experience*, 12, 23743735251314622.
