# Supplementary material for: Protocol for the Houston Hospital-based violence intervention program
Source: PLoS One. 2025 Jul 2;20(7):e0325569. doi: 10.1371/journal.pone.0325569 (PMC12221051; doi:10.1371/journal.pone.0325569)
Supplement: S1 Protocol — (DOCX) [file pone.0325569.s003.docx]

| **Protocol Title:** | Incorporation of a Health Equity Approach to Hospital Violence Intervention Programs: The Integration of a Community and Hospital Based Initiatives to Reduce Gun Violence in a Large Metropolitan Area |
| --- | --- |
| **Principal Investigator:** | Dr Sandra McKay and Dr Alexander Testa |
| **Co-Investigators:** | Drs Lillian Kao, Jack Tsai, Mary Aitken, Shreela Sharma, Vanessa Schick, Rousha Li, Kevin Rix, and Christian P. Owen |
| **Study Coordinator:** | Eresha Bluth |
| **Study Team:** | Mr Matthew Stephens, Ms Heidi McPherson, Ms Anne Marie Thompson, Ms Latanya Monroe |
| **Population:** | Males and female patients age 16-35 years who are victims of firearm injury, excluding self-harm, and unintentional injury |
| **Number of Sites:** | Single site – UTHealth/Memorial Hermann Hospital |
| **Clinical Trial:** | Phase III trial \| NCT06263647 |
| **Study Duration:** | 5 years |
| **Subject Duration:** | 12 months |

**General Information**

One of the most significant risk factors for violent injury is having a previous violent injury; thus, it is imperative to intervene with patients in the hospital setting to reduce the risk of reinjury through violence intervention programs. This study develops and implements the Houston Hospital-Based Violence Intervention Program (Houston-HVIP) in Memorial Hermann Hospital-Texas Medical Center. This study will evaluate the effectiveness of the Houston-HVIP in reducing firearm violence and improving recovery among young adults in urban trauma settings, thus ultimately improving community safety.

**Background Information**

Firearm violence is a leading cause of injury and mortality in the United States. ***Victims of firearm violence are often stuck in a cycle of violence, as they are twice as likely to die or be hospitalized from a violent event compared to those without a history of violent victimization***. Therefore, hospital-based intervention programs (HVIPs) can be crucial to provide holistic services and connections to critical community-based violence interventions that support firearm violence victims and reduce reinjury risk. Developing interventions to impact firearm violence requires a transdisciplinary approach with community–city–hospital interventions. The lack of a current HVIP in the greater Houston area–the fourth largest city in the United States–highlights the need to develop and implement this program as a critical community-based intervention to reduce firearm violence. Therefore, the current study aims to create the Houston-Hospital-Based Violence Intervention program at Memorial Hermann Hospital in the Texas Medical Center in Houston, Texas, as a community-level response to firearm violence. In Aim 1, during the UG3 phase, we will develop a coalition of internal and external stakeholders to advise on the formation and oversee the implementation of the Houston-HVIP. We will create the culture prime for the reception of the HVIP by developing an inter-professional, trauma-informed care educational platform. In our second aim, during the UG3 phase, we will implement and evaluate the Houston-HVIP program to determine the effectiveness of reducing repeat violent events by a program participant. The Houston-HVIP will feature a randomized controlled trial, with a high-intensity case management service program (Houston-HVIP) tailored to the firearm victim's needs, and standard of care program with referrals to community-based services. The program is guided by critical stakeholders, including hospital leadership, public health advocates, law enforcement, community and city leaders, and a multidisciplinary team of experts. This intervention will be rigorously evaluated to understand program effectiveness and acceptability, the impact of reinjury on those enrolled in each arm of the program, and the role of social determinants of health on firearm injury to the participant and the community.

**Objectives**

**Our primary aims for the proposed study are:**

**Aim 1: In the UG3 two-year planning period (2023-2025), our primary objective** is to develop a coalition of internal and external stakeholders to advise the formation and oversee the implementation of the Houston-HVIP program. In the planning period, we will create the culture prime for the reception of the HVIP by developing an inter-professional, trauma-informed care educational platform. Finally, we will develop the study evaluation framework, recruitment, retention, and measurement protocols and test the measures through a feasibility test of the intervention in preparation for the UH3 phase.

**Aim 2a: In the UH3 phase (2025-2028)**, we will conduct a randomized controlled trial to evaluate the effectiveness of Houston-HVIP in reducing the occurrence of repeat firearm violent events among children (16 and 17 yr olds) and adults receiving care at Memorial Hermann Hospital in Houston, Texas (*n* = 274); intervention group will receive the Houston-HVIP program, while the comparison group will receive standard of care with referrals to the community-based services.

**Our UH3 secondary aims are:**

**Aim 2b.** To determine the impact of the community-engaged Houston-HVIP program on violent re-injury and mental and behavioral health. We anticipate that those enrolled in the Houston-HVIP will have significantly less violent reinjury as measured by (a) return to the hospital for an intentional firearm and non-firearm injury, (b) self-reported firearm or non-firearm victimization, or (c) mortality from a firearm or non-firearm injury.

**Aim 2c.** To determine the impact of the community-engaged Houston-HVIP program on physical and behavioral health during the 12-month follow up period. We anticipate that compared to standard of care, those enrolled in the Houston-HVIP program will have significantly better health as measured by (a) PTSD [PCL-2], (b) Health and Wellbeing [SF-12], and (c) Aggression [Copeland-Linder].

**Aim 2d.** To identify the predictors of implementation success, including dosage, reach, fidelity, and acceptability from the perspective of gun violence victims, health care providers, and community violence intervention specialists**.** Data from quantitative surveys, and qualitative interviews of key stakeholders will inform an understanding of key features leading to the successful implementation of the HVIP program.

**Impact:** The proposed project brings together a transdisciplinary team to establish the first HVIP in the TMC. If found to be effective, the program has the potential to significantly reduce the incidence of firearm violence in Houston, Texas, and inform violence intervention efforts in other healthcare settings in the city and state. As a part of our study design, we are excited to participate in the Community Level Interventions for Firearm Prevention Research Network through this funding mechanism, thus contributing to the larger community in the advancement of firearm injury research.

**Study Design**

This five-year proposal will consist of a two-year planning period to develop and refine the Houston-HVIP procedures and train staff, thus instilling the culture transformation with a community-based approach and finalizing the recruitment, retention, measurement, and implementation protocols for the intervention delivery. This stage will be followed by three years focused on program implementation, data collection, and overall program evaluation.

**Aim 1: Develop a coalition of internal and external stakeholders to advise the formation and oversee the implementation of the Houston HVIP program.** In the planning period, we will create the culture prime for the reception of HVIPs by developing an inter-professional, trauma-informed care educational platform. Finally, we will develop the study evaluation framework, recruitment, retention, and measurement protocols and test the measures through a feasibility test of the intervention in preparation for the UH3 phase.

Using a community-centered approach, we will engage internal and external stakeholders to form a coalition that advises the implementation of the Houston HVIP. We will recruit members to include hospital leadership, community leaders, trauma specialists, law enforcement officers, city leadership, injury prevention specialists, previous victims of violence, and representatives of the Health Equity Collective to a goal of 15 total members. To convene, leverage and assess the coalition, we will undergo a series of modified intervention mapping steps to outline the scope of the Houston HVIP by focusing on steps 1-3 of Intervention Mapping (IM).^1,2^ The first step of IM requires the advisory board to conduct a thorough needs assessment, propose a logic model, and articulate program goals. Group facilitation techniques such as nominal group and brainstorming will play a crucial role in these meetings by encouraging active participation, open discussion, consensus generation, and problem-solving among group members. We will combine quantitative and qualitative research methods in Step 1. First, we will review the epidemiological analysis of firearm injury data obtained from the Memorial Hermann hospital system to estimate the prevalence and incidence of firearm injuries, specifically with a focus on firearm assault, in Harris County for the advisory board members. We will conduct a comprehensive review of relevant literature to identify the most critical and modifiable factors related to community violence and modifiable factors. The second step of IM is the foundation for our intervention by identifying who and what will change due to our efforts. The product of this step is a set of matrices at different ecological levels, including individual, community, and societal. These matrices will ensure that our intervention is comprehensive and inclusive in its community-based approach. In the last step, the objective is to confirm the scope and sequence of the program, develop a mechanism for feedback to the program leadership, and examine evaluation tools for comprehensiveness.

Led by Dr. Owen, we will develop an educational series on trauma-informed care to reach the critical health professionals and stakeholders involved in the Houston HVIP development and implementation. As we develop the culture of change, we will implement a series of educational interventions that will be guided by the Kirkpatrick model, an established model of analyzing and evaluating educational programs; we will evaluate the effectiveness of our educational programming at multiple levels. The core levels of the Kirkpatrick model evaluate the learner in their satisfaction of the education (Level 1), change in learning (Level 2), and change in behavior (Level 3) and impact to the population (Level 4).^3,4^ Each assessment of the level of learning will demonstrate the rigor of the educational process and the sustained impact on the learner.

This educational platform will include a series of asynchronous and live educational venues taught by experts in the field to transform the culture at Memorial Hermann Hospital and thus ensure adequate support for the planned intervention. Educational models will be developed by Dr McKay, Sharma, Aitken, Owen, and Mr. Harris, with core learning objectives of 1) Describing violence and risk factors, 2) Reviewing risk factors for recidivism, and 3) Roles of healthcare and communities to reduce gun violence. Dr. Owen will oversee module development to ensure alignment with goals. All educational events will be evaluated via a pre/post-test model (TIC Provider Survey v2.0 - All patient version), with assessments in knowledge, attitude, and behavior around core learning objectives obtained via quantitative surveys utilizing a series of Likert, yes/no, or sliding scaled answers before and immediately post-educational modules. Quantitative analysis will be performed via paired t-test or Chi-squared test, and additional statistical analyses will be used as appropriate.

Within our program, we will focus on identifying victims of firearm violence and presenting them to the Memorial Hermann Hospital at the Texas Medical Center during the study period. We will have two levels of patient support: 1) The Houston-HVIP program (Treatment Intervention) and 2) Standard of care (Comparison Group). Patients will be randomly assigned to either condition, thus supporting the ability to generate causal estimates of each treatment condition of the HVIP program on primary and secondary outcomes.

**Those in the high-intensity case management (Houston-HVIP) group will receive conditions #1-4 below:**

1. A case manager is assigned to meet with the injured patients to provide a brief assessment of psychological and social needs and individualized discharge planning bedside.
2. Coordinated referral, networking, and close follow-up with community-level violence outreach organizations from the City of Houston Mayor's Office and the Health Equity Collective that will provide outreach and counseling to victims, their family, and friends.
3. They will be referred to tailored social service programs based on the patient's needs, including assessments of social determinants of health, educational and financial needs, and subsequent referral for programs such as job training, educational support, housing assistance, or financial assistance programs for identified needs. Participants who are gang-related or violent perpetrators will be referred to the violence interrupter services with The Forgotten Third. Long-term opportunities for engagement with the trauma survivors' network within Memorial Hermann Hospital will be available and encouraged.
4. Participants in this program will receive services (either virtually or in-person based on the participant’s preference) characterized by intensity, complexity, and causal with an assigned case manager **over 6 months** to promote successful community-based program integration and completion: (a) **intensity** (≥8 participant contacts, biweekly or more frequent interactions, ≥30-minute encounters, 6+ months of follow-up), (b) **complexity** (addressing multiple social needs, involving multidisciplinary teams, providing active assistance with resource navigation, and tailoring services to individual needs), and (c) an emphasis on **enhancing causal inference** by through the use of an RCT.

**Those enrolled in the Standard of Care Program will receive the conditions #1-3 below:**

(1) A case manager is assigned to meet with the injured patients to briefly assess psychological and social needs and individualized discharge planning bedside.

(2) Resources for the patient to connect with social service programs based on the patient's needs, including assessments of social determinants of health, educational and financial needs, and subsequent referral for programs such as job training, educational support, housing assistance, or financial assistance programs for identified needs. Long-term opportunities for engagement with the trauma survivors' network within Memorial Hermann Hospital will be available and encouraged.

(3) Follow-up contacts and services from an assigned case manager **over two weeks** following discharge from the hospital to ensure that the patient has contacted the community-level violence outreach organizations.

Those who decline enrollment will receive usual hospital care and are offered services via the hospital-based social worker. The typical standard of care under current hospital guidelines includes the following conditions: stabilization for a current medical condition, referral to the hospital social worker for assessment of social concerns, and referrals for services while the patient is admitted to the hospital.


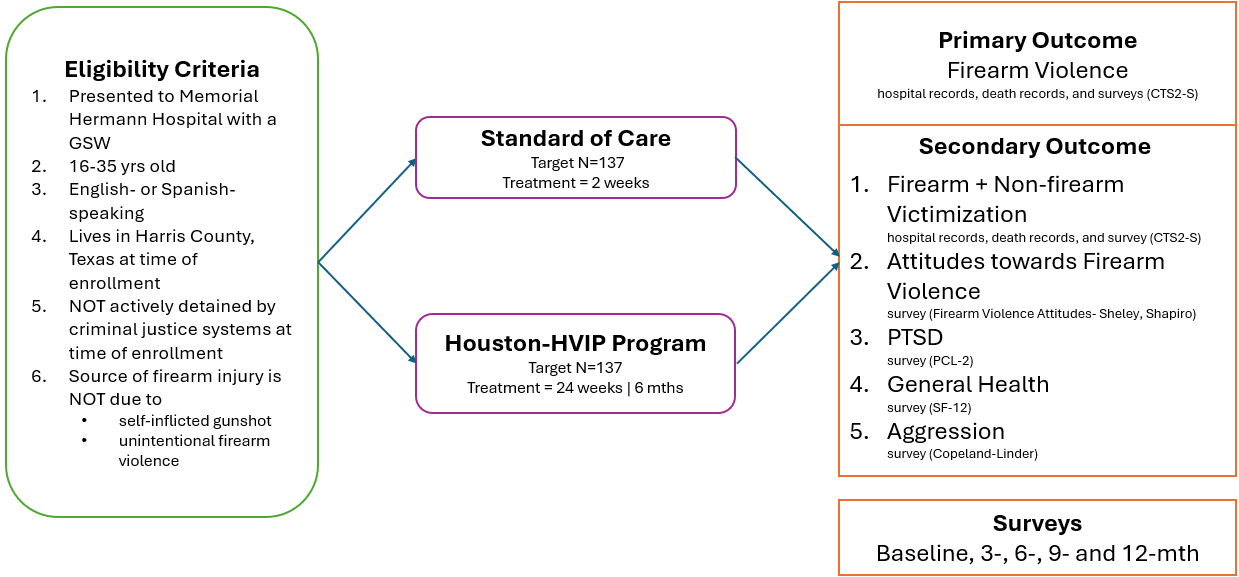
**Study Flow**

**Evaluation of UG3 Phase Activities:** Coalition meetings will be conducted in-person and virtually to accommodate all participants, while thorough note-taking ensures no loss of input. Summaries of these meetings are shared with the Community Advisory Board (CAB) members in a timely manner. One or more investigators will lead the groups (McKay, Testa, Sharma). Participants will be compensated $50/meeting for their time. Data captured in the process will be secured on password-protected storage drives at UTHealth for ongoing data management, and Institutional Review Board approval will be obtained. We plan to hold six meetings prior to the pilot phase and at least 2 virtual meetings after the initiation of the pilot group to provide an update to the group.

**Qualitative Analysis:** We will conduct semi-structured virtual interviews with coalition members who volunteer to do the interview. These interviews will be audio-recorded and professionally transcribed. Participant names will be removed during transcription. A multidisciplinary research team will use a four-step approach to analyze qualitative data. First, the team will collectively read the transcripts collected from each interview to better understand the general themes. Through this process, a deductive codebook will be created to allow labeling of the text from the interviews. These codes will be generated in group analysis sessions until the team has agreed on all codes. Second, the team will code all the text. We will group emerging findings into categories of themes using an immersion-crystallization approach, including inductive thematic identification.^5^ Third, transcripts will be read by a second coder, and coding inconsistencies will be discussed and resolved by consensus between the two coders. Fourth, the team will consider how the findings relate to the current health literature, health services research, and intervention development.

**Quantitative Analysis:** Surveys will be conducted with coalition members to assess the feasibility and acceptability of aspects of programming, consisting primarily of Likert ratings. To determine the impact of program design, we will conduct an analysis of variance (ANOVA) based on the portion of the program being evaluated. Other analysis approaches may be incorporated if appropriate.

**Mixed-Methods Analysis:** Mixed-method findings will provide a more nuanced understanding of the effect of implemented strategies proposed in the toolkit. Analyses for each data collection method will be conducted independently to ensure unbiased interpretation. After completing those analyses, qualitative and quantitative findings will be integrated using data-triangulation techniques.^66^ Triangulating such data strengthens validity and decreases deficiencies or biases that might arise from any single method.^6–8^ Our multidisciplinary research team minimizes researcher bias by bringing experts with differing perspectives to review and critique the synthesis and interpretation of qualitative findings.

**Pilot Phase:** The ultimate assessment of the foundational work of the UG3 phase will be to perform a pilot phase of the Houston-HVIP, thus ensuring that the development of the intervention is complete and all aspects of the program are functional to the best of its capability. This will allow feedback to the coalition to enable program modification before a larger-scale implementation trial. First, all necessary agreements, operations manuals, and process flow maps will be developed and approved, and study materials, including computerized study surveys, will be created. Second, ten patients will be recruited to participate in the pilot Houston-HVIP over three months and will be randomly assigned to each condition. Patients and case managers will be interviewed after three months after recruitment for the feasibility and acceptability of the model. They will undergo semi-structured interviews in addition to the routine assessment of the Houston-HVIP. Semi-structured interviews will be evaluated as described under qualitative analysis. Results of this feasibility testing will be used to finalize the intervention and evaluation design and delivery of the UH3 phase.

**UG3 Milestones.** For a successful transition to the UH3 phase of the program, we will define milestones for critical aspects of the program. First, we expect full integration of trauma-informed care education to the hospital and staff, with at least 80% of eligible staff completing the educational module successfully. We will continue to educate qualified personnel until we have reached our goal and will leverage the coalition if needed for institutional support. Secondly, we will convene the coalition with the completion of the IM steps. These steps will serve as the metric for success as they will outline the formation and implementation of the Houston HVIP. The Health Equity Collective will serve as a support, and the Center for Health Equity will guide the IM framework to ensure completion. Finally, with the help of the coalition, completing a pilot with ten patients to demonstrate the feasibility and acceptability of the program will further show that the project is ready to move into the UH3 phase. By the end of the pilot phase, we will finalize all protocols for measuring and implementing the intervention.

**Aim 2: In the UH3 phase (2025-2028), our primary objective** is to conduct a randomized controlled trial to evaluate the effectiveness of Houston-HVIP in reducing repeat firearm violence events among adults receiving care at Memorial Hermann Hospital in Houston, Texas. This stage will include the random assignment of individuals to the primary intervention of the Houston-HVIP program or the comparison group that will receive the standard of care. A key aspect of this intervention will be assessing the impact of the Houston-HVIP program on violent re-injury and mental and behavioral health. Finally, we will aim to identify the predictors of implementation success in our model from each critical stakeholder perspective.

**Study Design.** The current study will focus on individuals that present to Memorial Hermann Hospital at the Texas Medical Center during the study period for community-based intentional firearm injury. The eligibility criteria for the study will be individuals aged 16-35 who presented for a gunshot injury stemming from community violence at Memorial Hermann Hospital, are English or Spanish-speaking, and provide informed voluntary consent or parent’s informed consent and child assent for the minor participants to participate in the study. Exclusions will be for individuals outside of this age range (i.e., below 16 or over 35 years old), those presenting for a firearm injury arising from a self-inflicted gunshot or unintentional gun violence, those declining voluntary informed consent, those who live outside of Harris County, those who are actively detained by the criminal justice systems at the time of enrollment, and for patients who speak a primary language that is other than English or Spanish, due to capacity limitations from community partners.

**Recruitment Plans:** Recruitment will occur following patient intake, and recruitment procedures will occur seven days a week. We will recruit individuals into the study and randomize them to the intervention and comparison conditions. The targeted enrollment for **the Houston HVIP program** and Standard of care are 17-18 patients per month during the recruitment window to a total of 137 (in each program). The anticipated total enrollment for both arms is 274 participants. Once target recruitment goal is achieved, enrollment will cease. Because the program is a 1 year intervention and the grant cycle for the UH3 phase is 3 years, we estimate needing 6 months after final participant has completed the program to complete data analysis. As a result, we anticipate a total recruitment window of 18 months.

Eligible patients will be identified from electronic medical records and integrated trauma databases within the hospital system to allow for real-time notification of admission to the Memorial Hermann Hospital and approached by program staff before discharge. This procedure will allow the recruitment of patients treated for gunshot wounds which are not critically injured and thus need hospital admission and eventual discharge from the hospital. This integrated notification system and a 24/7 recruitment process will allow research staff to approach and identify eligible patients for the study. The clinical team will also be provided with cards that includes a QR code (linked to the study website) and can also refer patients, and program staff will screen for eligibility. Once patients are deemed eligible, staff members will physically engage with patients at the hospital bedside to establish trust and assess the needs of the patient. The research team will coordinate with the treatment team to ensure that they are not interrupting patient care, and that timing is appropriate (i.e., not approaching a patient who is unstable or critically ill). Participation in the program will be contingent on obtaining voluntary informed written consent from the patient.

For patients who meet eligibility criteria but are discharged before engaging with a case manager at the bedside, the case manager will make up to eight follow-up attempts over two weeks using the contact information in the EMR. The case manager will attempt to reach individuals via telephone at various times and on different days of the week. Patients who decline participation at any time are no longer contacted. If a discharged patient expresses interest in participating after being contacted, arrangements will be made to meet them in person at the hospital or virtually via Microsoft Teams, Zoom, or a telephone call to discuss study details and obtain informed consent via the REDCap system. The consents will be sent to prior to the meeting to allow sufficient time for the patient/parent (for the 16 and 17 year olds) to review the consent form. UTHealth’s “Remote Consent Process and Electronic Consent Signature” procedures will be followed.

**Recruitment and Retention Plans.** We will train case managers in motivational interviewing to increase the probability of participation. Individuals will also receive a financial incentive to compensate for their time. Our training will support a patient-centered approach within the hospital system with the goal of enhancing the overall implementation and acceptance of the program. To mitigate high attrition from the study, we will use several evidence-based strategies for study retention, including interview incentives at baseline and follow-up periods, regular phone check-ins sent via text message to maintain contact, using a list of family and friends who can help maintain contact, and working closely with community partners to help maintain contact with the participant. These procedures were shown to lead to a 91% retention rate in a study of recently released prisoners in Boston over a 12-month period.^9^

**Randomization Procedure:** Participants will be randomly assigned to either the treatment or control condition using the randomization module in REDCap. After determining eligibility and obtaining informed consent, the case manager will update these participants’ status in REDCap, where the randomization process will occur.

The randomization scheme will be generated and then uploaded into REDCap under the randomization module to ensure equal allocation to the treatment and control arms. Specifically, the randomization module will utilize a blocked randomization procedure with varying block sizes to ensure that the number of participants in each group remains balanced throughout the enrollment process. The REDCap system will automatically assign participants to either the treatment or control condition based on this scheme, ensuring allocation concealment. The intervention will be administered at the individual level. This study will require voluntary, informed written/e-consent from the adult patient and from the parent(s)/LAR and an assent for the minor patient. (ages 16 and 17). Individuals who do not provide voluntary informed written consent/e-consent will not be included in either the treatment or comparison group and will not be included in the study. Once an individual participant eligible for enrollment is selected, a designated case manager can log in to the secured REDCap system and obtain a computer-generated randomization assignment in a timely manner. Authorized, unblinded individuals who need this information to provide the appropriate intervention successfully will receive an automated notification. Due to the nature of the proposed intervention, case managers will be unblinded regarding the group assignment but will be blinded to the outcome data. Participants will be partially blinded in that they will know some information about the intervention but will not be exposed to the details of the intervention or the intervention manual. The study personnel who collect or evaluate outcome data will be blinded to the intervention. Data will be collected and stored on a readily accessible dashboard hosted through REDcap and maintained by the School of Public Health throughout the project.

**Intervention Implementation.** The intervention will be implemented during the UH3 phase using a fully powered RCT with eligible participants assigned to the Houston-HVIP program or the standard of care comparison group. Ultimately, coalition members will provide input on the intervention implementation and delivery through surveys and focus groups to provide guidance on the feasibility and acceptability of the program components.

**Quantitative Data.** Data for this study will be collected through three primary sources: (1) hospital records, (2) baseline survey before hospital discharge (baseline), 3-, 6-, 9- and 12- month follow-up surveys through program completion and (3) interviews. They will receive an electronic $50 gift card upon completing the first survey and $100 for each of the follow-up surveys (3-month, 6-month, 9-month, and 12-month). Options to take the follow-up surveys at UTHealth facilities or through the community partner will also be provided. The list of variables to be included in this study is described in Table 3. We will track our primary outcome of repeat firearm violence and victimization.

**Aim 2a: To determine the impact of the community-engaged Houston HVIP program on firearm violence during the 12-month follow up period.** Our primary outcome for Aim 2a is measures of repeat firearm violence as measured by (a) return to the hospital for an intentional firearm injury, (b) self-reported firearm victimization, or (c) mortality from a firearm injury. We will use hospital admission records to identify individuals who return to the hospital system for a violent injury during the study period. Second, to capture those victims of violence that do not result in hospital readmission, we will use self-reported measures of firearm violence during the follow-up period captured using the REDCap survey. Third, we will measure mortality from a firearm injury from death records. To achieve **Aim 2b: To assess the impact of the Houston-HVIP program on reducing violent reinjury**, we will assess (a) hospital readmission data covering both adult Level 1 trauma centers in the Greater Houston area, (b) comprehensive national death record searches, and (c) self-reported information using a validated instrument.

**Aim 2c: To determine the impact of the community-engaged Houston-HVIP program on physical and behavioral health during the 12-month follow up period.** We anticipate that compared to standard of care, those enrolled in the Houston HVIP will have significantly better health as measured by (a) PTSD [PCL-2], (b) Health and Wellbeing [SF-12], and (c) Aggression [Copeland-Linder].

**Aim 2d. To identify the predictors of implementation success, including dosage, reach, fidelity, and acceptability from the perspective of gun violence victims, health care providers, and community violence intervention specialists**. Data from quantitative surveys and qualitative interviews of key stakeholders will inform an understanding of key features leading to the successful implementation of the HVIP program.

**Semi-Structured Interviews.** To understand the mechanism of the program and for a process evaluation, we will recruit a subset of patients enrolled in both the Houston-HVIP and standard of care Program conditions after the study period (*n* = 20 from each group) to conduct semi-structured interviews. This sample will provide adequate consultations from each treatment group to reach saturation.^10^ We will also conduct semi-structured interviews with n = 20 healthcare providers, community violence specialists, and case managers. The purpose of these interviews is to achieve Aim 2c: To identify the predictors of implementation success, including dosage, reach, fidelity, and acceptability from the perspective of gun violence victims, health care providers, and community violence intervention specialists. Semi-structured interviews with one or two research team members will last approximately 60 minutes and will be conducted virtually or telephonically three months [pilot group] / fifteen months [full study trial group] after the initial hospitalization. Researchers will use an interview guide composed of open-ended questions to structure the interview. Open-ended questions will allow unanticipated themes and issues to emerge and allow the participants to elaborate on their individualized experiences in their own words. Interviews will be audio-recorded and transcribed, and analyzed using thematic analysis similar to the UG3 section under qualitative analysis.

**Sample Size and Statistical Power.** We base our sample size calculation on a comparison of firearm violence between two randomization groups (Houston HVIP vs. SOC) with a two-sided alpha of 0.05. The primary outcome of firearm violence is a composite of (a) return to the hospital for an intentional firearm injury (hospital records), (b) self-reported firearm victimization (CTS-2), or (c) mortality from a firearm injury (mortality records). We will follow the participants at 3, 6, 9, and 12 months following the baseline visit and record whether the outcome occur between the visits. We will compare time to firearm violence between the two arms using a Weibull regression model for interval-censored data to account for the interval-censoring and potential loss to follow-up. A prior study among youth seeking assault-injury care at an emergency department observed a two-year rate of 59% for this composite outcome, where the reporting rates between 0-6, 6-12, 12-18, and 18-24 months were 37.5%, 25.8%, 20.5%, and 15.2% respectively^11^. This pattern suggests a potential decreasing hazard over time that can be well accounted for by a Weibull distribution, which flexibly allows for increasing, decreasing, or constant hazard shapes. We conducted simulations based on a Weibull distribution with a two-year probability of 59% in the SOC arm, corresponding to a one-year probability of 42%. To estimate the power, we generated 10,000 simulated data from this distribution, imposed interval-censoring according to the planned follow-up, added right censoring from potential attrition (patient lost to follow-up), and applied the planned Weibull regression model. Under a sample size of 274 (137 in each arm) and allowing up to 35% attrition during the follow-up, we have 80% power to detect a hazard ratio (HR) of 0.50, corresponding to a reduction from 42% to 24% in one year. The planned adjustment of important risk factors in the Weibull regression model will offer enhanced power. The planned sample size will also provide adequate power for the secondary outcomes. For example, for a continuous outcome such as SF-12, a complete sample size of 200 (100+100) allows us to detect a small to moderate between-group difference, Cohen’s d=0.40, with 80% power and a two-sided alpha=0.05 in a two-sample t-test. This calculation is conservative as the repeated measure for these outcomes provide enhanced power in the planned mixed effect model when compared to a two-sample t-test at one time point.

**Statistical Analysis.** Primary analysis will follow the intention-to-treat principle. A Weibull regression model for interval-censored data will be used to compare the risk of firearm violence between the two study groups. A two-sided alpha of 0.05 will be used to determine statistical significance. Since prespecified adjustment of important baseline covariates can provide enhanced power, the regression model will be adjusted for race/ethnicity, biological sex, and age. In addition, we plan to balance-cross case managers across the two arms to protect against potential agent effects, if any, and will adjust case manager as a fixed factor covariate in our analytic model^83^. We will carry out several secondary analyses to gain comprehensive insights and ensure the robustness of findings. Although the random enrollment of participants to the two treatment arms should ensure comparability with respect to known and unknown variables, imbalance may occur by chance. Descriptive statistics for baseline characteristics known or suspected to be associated with outcomes will be prepared for the two randomization groups. Chi-square statistics and Wilcoxon rank sum tests will be used to evaluate baseline differences between the arms for categorical and continuous variables, respectively. Any variables with baseline differences will be adjusted in secondary sensitivity analyses. Next, to assess heterogeneity of the intervention effect, we will examine interactions between the randomization group and important baseline risk factors (e.g., race/ethnicity). The corresponding interaction terms will be added individually into the regression model to examine potential effect modification. Finally, we will carry out a per-protocol analysis as a sensitivity analysis.

The secondary outcomes that are measured longitudinally (e.g., SF-12) will be analyzed using appropriate longitudinal models, such as the (generalized) linear mixed model with a suitable distribution and link function, including group, time (categorical), group by time interaction, as well as subject-specific random effects to account for the repeated measures. We will first conduct a contrast to test the intervention effect across the post-baseline time points. Group-specific means and between-group differences will be estimated at each time point with 95% confidence intervals.

In all statistical analyses, we will conduct careful checking regarding all model assumptions to ensure adequate model fit. For example, we will examine the fit of the Weibull regression model by comparing the model-based estimates to its empirical counterpart across the time-intervals (0-3, 3-6, 6-9, 9-12 months) by arm. We will also check the residual normality, homoscedasticity, and linearity for the linear mixed model. Necessary remedies will be implemented to ensure adequate model fit. We will adopt rigorous methods for handling missing data, by first examining the degree and patterns of missingness and identifying factors that affect the missing rates. We will adopt appropriate statistical methods, such as multiple imputation and inverse probability weighting, under plausible assumptions about the missing mechanism.

**Research Design Considerations.** There are possible threats to recruitment, which the study team has anticipated and has put into place plans to handle. *Individuals not wanting to participate*: To maximize participation, all staff involved in recruitment will be trained in motivational interviewing to increase the likelihood of participation. Individuals who opt to participate in the program will also be given a financial incentive to provide compensation for their time. In cases where individuals refuse participation in the program, we will ensure standard care is provided to ensure some services are rendered.

**Study Population**

Participants will be recruited from Memorial Hermann Hospital patients aged 16 to 35 who presented for a gunshot injury from community-based firearm violence. This age range is selected to capture those with the highest risk for violent victimization incidents. Exclusions will be made for self-inflicted gunshots as the source of firearm injuries.

**Eligibility Criteria**

We will include victims of firearm violence presenting to Memorial Hermann Hospital during the study period, who are English and Spanish speaking and between the ages of 16-35 years old. Exclusion criteria are if they are under the age of 16 or above the age of 35, presenting for a firearm injury that is the result of a self-harm attempt, or unintentional injury, lives outside of Harris County at the time of enrollment, actively detained by the criminal justice systems at the time of enrollment, or if they are non-English (other than Spanish) or non-Spanish speaking(other than English) (meaning we will only include patients who can speak either English or Spanish).

**Age limits**

Min: 16 Max: 35

**Inclusion of individuals across the lifespan**

Our study population will be victims of firearm violence, ages 16 to 35 years, as these are the ages of highest firearm violence, as indicated by data from Memorial Hermann Hospital. We will also identify special populations, including parents, women, minorities, or active duty military/veterans, through our data collection process. We will also exclude incarcerated individuals.

**Inclusion of Women and Minorities**

With the written informed consent from the parents/LARs and child assent, we will enroll children ages 16 and 17 as they have one of the highest rates of firearm violence. We will enroll both male and female study participants. However, we recognize that firearm violence is more common among males and thus the study sample is expected to skew heavily toward males. In addition, we will recruit across all racial and ethnic groups. However, because firearm violence is more common among African American and Hispanic persons relative to White individuals, we anticipate the sample to be more highly composed of Black and Hispanic persons, and anticipate no challenges related to being able to recruit a sample inclusive of minority populations.

We plan to recruit participants from Memorial Hermann Hospital who meet eligibility criteria and who consent to participate in the study according to the study timeline. All initial study contacts will be done via research staff during the hospital presentation. Case managers will provide regular contact using motivational interviewing techniques to maintain enrollment in the program. Gift cards will be utilized to incentivize study retention.

**Data and Safety Monitoring**

Any potential risk to the participant will be minimized by strictly adhering to the research protocol, as approved by the IRB at UTHealth and DHHS for the Protection of Human Subjects. Participants will be informed of the study and risks to participants and will give consent before participation. Participants will be informed that this is voluntary and they can withdraw from the study anytime. No physical or psychological adverse effects of the intervention are known or anticipated. There is minimal incremental risk beyond the risks that are associated with the individual components of the HVIP and enhanced services.  Research related risks are limited and include time spent completing the surveys and potential distress caused by completing the survey.  Processes are in place for study participants to discuss any distress with their case manager, and they can be referred to community support services as needed.

Risks to Human Subjects: All subjects will give informed consent before study enrollment.

Adequacy of Protection Against Risks: Research staff will undergo training in informed consent, and the survey will feature written informed consent before initiating the survey. All aspects of the study will receive IRB approval before initiation. This study does not involve fetuses, or neonates. We will not be asking about pregnancy status in this study.

Potential Benefits of the Proposed Research to Research Participants and Others: There are potential benefits to the participant in the study, with the enhanced services in case management and connection to community-based resources. Studies from previous HVIP interventions have demonstrated significant decreases in re-injury, and thus there is potential for decreased violent re-injury to the patient enrolled in the study.

Importance of Knowledge to be Gained: Understanding the benefit of HVIP to the community and its role in implementing HVIP will be important to the larger firearm violence research networks as a whole. The results of this program can serve as a model of other programs established in hospital systems as a means to curtail violence. If successful, the program can be maintained in the hospital system and will continue to yield benefits in reducing violence in Houston after the study period has ended.

A Data Safety and Monitoring Board (DSMB) will be convened for this study. The DSMB will review the study protocols and provide oversight of recruitment progress, data quality and completeness, efficacy monitoring, and participant safety as it pertains to the study. The research team will be responsible for providing study protocols, data and adverse event reports to the DSMB. All recruitment and data collection will be conducted in Houston, Texas. A formal independent board of experts including investigators and at least one biostatistician will be appointed to the DSMB. The Board will meet annually (at a minimum) to review adverse event data, other safety data, quality and completeness of study data, and enrollment data to ensure proper trial conduct. Our study team will provide any new literature particularly pertinent to the trial, along with their recommendation as to whether it affects the trial conduct or design. The DSMB will review the consent form at regular intervals to determine whether the consent form requires revision in light of any new findings or amendments. All events will be evaluated by the DSMB in accordance with its charter and review procedures (which will be reviewed with the Chair and Members at the initial meeting). This includes an assessment of expectedness, relationship to the study product or procedures, and severity. The study investigators will rely on the DSMB to identify conditions or events that would trigger further action, including a temporary halt, modification, or termination of the study for safety reasons.

**Data Management**

Drs. McKay and Testa and study investigators will meet weekly with research staff to discuss the research study's day-to-day operations, minimize risk, protect participant data's confidentiality, and report adverse events and unanticipated problems. Drs. Testa and McKay and study investigators will ensure and monitor study safety with rigorous training and supervising staff in the field. Participant data will be protected by using unique IDs for all participants and a secure database for data collection.

**Adverse Events**

For the purpose of this protocol, an Adverse Event (AE) is any undesirable or unintended clinical occurrence in a subject that can be attributed to the study intervention. Adverse events may be expected or unexpected. In addition, some AEs may meet the definition of Serious Adverse Event (SAE).

**Expected Adverse Events**

An expected adverse event is an AE for which the nature, severity, or degree of incidence is known and identified in the study protocol. Expected adverse events associated with this study include:

**1) Breach of confidentiality** associated with data collection and necessary reporting of increased participant risk (suicide risk, homicide/retaliation risk, or risk based on increased substance use) to appropriate authorities or health personnel.

There is a minor potential risk to the confidentiality of survey data and audio-recorded (via UTHealth Teams) sessions. Precautions to protect subject confidentiality will be maintained through the use of study identification numbers and data stored in password-protected files on UTHealth password protected servers. All personnel assisting in data collection have been trained to perform required tasks safely, effectively, and efficiently and maintain patient confidentiality. Only the principal investigator, co-investigators, and necessary research personnel will have access to this data. Study staff will have procedures in place to ensure data confidentiality.
 **2) Homicidal ideation/retaliation risk** as indicated by verbal report of thoughts of hurting others.

We conduct an assessment of retaliation risk given that the initial injury occurred during a violent altercation. Further, homicidal or retaliatory thoughts may also be provided by the participant during an interaction with study team members. The protocol for handling homicidal ideation or retaliation risk will be similar to that for suicidal ideation. Specifically, if the participant articulates thoughts of hurting others, the study team member will ask the participant to elaborate on his/her thoughts/behaviors (e.g., is there an identified victim, does the participant have a plan). The level of action necessary will be based on the participant’s responses and whether the study team member perceives that the patient or someone else is in immediate danger. If a participant espouses any retaliatory ideation during subsequent interactions with staff, a homicidal ideation screener will be provided to the participant by a case manager to assess risk. Study personnel will follow established safety protocols and contact local licensed clinical staff when appropriate.

Our team has extensive experience studying high-risk patients and have concrete plans to manage potential risks, including suicide risk, retaliatory risk and/or disclosure of child abuse. Briefly, all participants will be informed during consent that we will need to inform a mental health provider if they are determined to be at significant risk and/or if they disclose information regarding risks during sessions or in the course of study procedures. Measures at baseline/follow-up will assess suicidality and retaliation risk; they will flag if endorsed, prompting formal risk assessment. A determination of high-risk status will be made through review of these measures, the formal risk assessment criteria, and discussions when appropriate with a licensed clinician (e.g., Dr. Tsai). Whenever possible, if a participant is determined to be at high acute risk, we will engage them in the process of disclosing this to a provider (at baseline enrollment) and/or aid in contacting them with appropriate resources (during the intervention or at follow-ups). We will only break confidentiality if it is determined that it is necessary to manage the acute risk (e.g., homicidal/retaliatory threats) and the participant is unwilling to engage in this process.

Drs. McKay and Testa will provide supervision to research staff who have direct contact with patients, including crisis procedures for suicide and homicide risk and clinical assessment and referral procedures for participants who meet threshold criteria for suicide or homicide. Supervision will focus on procedures for managing issues that could arise given the patient population, including potential crisis situations and/or adverse events around violence and/or substance use. Procedures will include a review of the study protocol regarding the limits of confidentiality, how to liaison with study site staff (i.e., psychiatry, crisis management) to arrange for an assessment, circumstances in which it may be necessary to notify parents/authorities regarding intent to harm self or others, and the development of safety plans and resources. The crisis procedures also will include immediately notifying Drs. McKay and Testa for consultation and conference call discussions on any risk assessments that meet more than minimal risk. Other investigators will be involved as necessary, and a 24/7 study staff member and investigator will be on call to manage crisis calls by participants at all times.

**3) Hospitalization or Emergency Department/Urgent Care visits** as a result of mental health (e.g., suicide attempts), substance use (e.g. acute intoxication, acute withdrawal), or violent injury (e.g., gunshot wound, assault injury).
 **4) Severe Emotional Distress**a. patient verbal report of a significant increase in psychiatric symptoms;

b. patients reporting significant distress due to the content of the self-report measures;

c. patients reporting significant distress due to the content of the case management sessions.

d. patients reporting significant distress due to talking about or reliving the experiences of the traumatic event.

Participants will be informed during the consent process and again at the start of each interview that they are free to “skip” any interview question or prompt that makes them uncomfortable and free to end their participation in the interview at any time. They will also be provided with information to contact the study MPIs and UTHealth IRB to discuss any concerns they might have about the study. Dr. McKay and Dr. Testa and several Co-Is (Tsai, Schick) have experience conducting these types of interviews with individuals who have a history of trauma. All study staff members who conduct in-depth interviews and interact with the study population will be trained beforehand in trauma-informed care to be sensitive to signs of participant discomfort or distress. Participants will be provided with information about available mental health resources throughout the duration of the study. Available mental health resources and mental health resources at Memorial Hermann Hospital will be made available to all participants.

In each case, the case manager and study staff will consult with the PIs to determine whether the participant’s distress was significant enough to warrant reporting as an AE.
 **5) Suicidal ideation**

a. self-report of significant suicidal ideations and behaviors on pertinent study surveys

b. endorsement of recent suicidal thoughts, plans, or actions during the assessment or intervention sessions

c. endorsement of thoughts of suicide or suicidal behaviors during the assessment or intervention sessions.

In each case, the case manager and study staff will follow the suicide risk assessment protocol and consult with the PIs to determine whether the participant’s distress was significant enough to warrant reporting as an AE.

**6) Psychological discomfort** as a result of being asked personal questions, particularly during the assessments and sessions. While we do not anticipate significant psychological distress during study, we will monitor reported distress during interviews so that participants can receive additional assessment and referrals as needed. If signs of discomfort or distress are detected and reported by interviewers to the study team, we will notify the participant. All participants will also be provided with information about available mental health resources and mental health resources at Memorial Hermann Hospital will be made available to all participants.

**Unexpected Problems (UP)**

An unexpected adverse event is an event that is not listed as a risk in the protocol or consent forms, or was not previously documented/observed in our study population. For an event or information to be considered an “unanticipated problem,”' three criteria must be met:

1. It must be **“unanticipated.”** This means the event is not expected in terms of its nature, severity or frequency given the:

o Procedures described in the study documents (e.g. the application, protocol, data and safety monitoring plan, etc.)

o Characteristics of the subject population being studied (the traits, behaviors, symptoms, diseases, life experiences, or other qualities typically found in the persons comprising those eligible to participate in the study). A UP is a problem that was expected by neither the research participants nor the investigators.

1. It must **be “related to the research.”** This means there is a reasonable possibility that the event or information may have been caused by, or is linked in a significant way, to the research. This encompasses all aspects of the research; it is not limited to test agents or procedures.
2. The event or information suggests that the **research places subjects or others at greater risk of harm than was previously known or recognized.** This includes physical, psychological, economic, or social harm:

o **Type 1:** Potential harm - Possibility that previously unsuspected harm may occur (or may occur at a higher than expected rate) even though no one has yet experienced actual harm.

o **Type 2:** Actual harm - Recognized harmful or unfavorable outcome that has actually occurred to a research subject, a set of subjects, another individual being treated in a similar fashion in a relevant non-research setting, or another person connected to the research study.

**Serious Adverse Events**

A Serious Adverse Event (SAE) is any untoward occurrence or effect that:

- Results in death
- Is life threatening
- Requires inpatient unplanned hospitalization or prolongation of existing hospitalization
- Persistent or significant disability/incapacity
- An important medical event that may not result in death, be life threatening, or require hospitalization may be considered an SAE when, based upon appropriate medical judgment, the event may jeopardize the subject and may require medical or surgical intervention to prevent one of the outcomes listed in this definition.

More than one of the criteria above can be applicable to one event.

***Death***

A determination of death must be made in accordance with accepted medical standards.

***Life-threatening***

The definition refers to an event in which the patient was at immediate risk of death at the time of the event.

***Hospitalization***

It is defined as inpatient care of more than one calendar day (overnight admission).

**Recording and Reporting Adverse Events**

At each evaluation, study staff (case manager, program manager, and/or graduate research assistant) will determine whether any adverse events (AEs) have occurred. All AEs that occur between enrollment and subject exit will be documented in a timely manner. As the PIs, Drs. McKay and Testa will have the ultimate responsibility for monitoring the overall safety of the participants in the study, determining whether an event is considered an adverse event (AE) or serious adverse event (SAE), and reporting events as needed to the appropriate committees and agencies. Study personnel will be expected to notify the lead PI, Project Manager, or other designated coordinating center personnel of serious adverse events, noncompliance, or other study related problems as soon as they are identified. In all instances where participant safety is a concern, the participant will be evaluated and connected with the appropriate level of treatment services necessary. Senior clinicians (Drs. Lillian Kao and Jack Tsai) are always on call for consultation if a participant seems distressed or needs special attention for medical or mental health-related issues. All participants will be instructed on how to obtain emergency care, should that be required.

**Data Safety Monitoring Board:**

A Data Safety and Monitoring Board (DSMB) will be convened for this study.

The Co-Principal Investigators (PI) will be responsible for ensuring participants’ safety on a daily basis. In addition, the study will impanel a Data and Safety Monitoring Board (DSMB) to act in an advisory capacity to the PI and to evaluate the progress of the study, including periodic assessments of data quality and timeliness, participant recruitment, accrual and retention, participant risk versus benefit, performance of trial sites, and other factors that can affect study outcome.  The DSMB will make recommendations to NINR’s Director concerning the continuation, modification, or conclusion of the trial.

Roles and Responsibilities: The DSMB will review study protocols and provide oversight of recruitment progress, data quality and completeness, efficacy monitoring, and participant safety pertaining to the study. The research team will be responsible for providing study protocols, data, and adverse event reports to the DSMB. All recruitment and data collection will be conducted in Houston, Texas.

DSMB Membership and Affiliations: The DSMB will be comprised of 5 individuals external to the grant to provide safety monitoring and oversight. All members of the DSMB will be external to the grant.

Frequency and Types of Meetings:

The DSMB will meet annually (at a minimum) via teleconference to review the study progress, data quality, and participants' safety.  During these meetings, the PIs and Co-Is will provide the DSMB with an update on study recruitment and enrollment, the progress of participants within the study, and the presence of all AE/SAE, including classifications based on severity, expectedness, and relatedness to study procedures.  The DSMB will then have private discussions and provide recommendations as appropriate.

**Data Safety Monitoring Plan Overview**

A detailed Data and Safety Monitoring Plan will be submitted to UTHealth’s IRB and to NIH prior to the recruitment of participants. The functions of the DSMB include (1) review and approval of the plan for data and safety monitoring; (2) review of protocols and questionnaires (3) review of recruitment procedures; (4) review of data, to ensure proper conduct and progress of the study; (5) review of credentials of investigators, project staff and consultants; (6) provision of recommendations to project investigators and staff regarding issues of concern or adverse events; and (7) review of potential ethical issues involved with the research.

Any changes to the protocol will be submitted to the institutional IRB at UTHealth. No changes will be commenced without the approval of this organization.

Direct data collection will begin at the time of screening and continue until it has been determined that the subject is not eligible for this trial, the subject or family refuses to consent, the subject drops out of the study, or completes the study. Data on eligibility will be submitted to the Data Management Team to allow a description of screened versus enrolled subjects. Data collection personnel will receive training on data collection, recording, and data management procedures. Procedures to ensure adherence to study protocols and data collection procedures include initial training, training reinforcement, and random checks. Data will be collected on consenting subjects using standardized case report forms. After data collection, the data will be entered into a secure, HIPAA-compliant, secure web-based data system designed for this trial. The web-based program provides the flexibility of entering data from multiple locations and centralizes the data management process. To ensure security, each user will be assigned a username and password and this username, date and time of each login is recorded in a login history file to ensure a record is maintained of each access to the system. This information will also be recorded in the change history audit logs. Data entry programs will require legitimate data formats, determination of required fields, key fields, range edits, value edits, and consistency checks involving two or more fields. Data must pass all quality control edits before acceptance into the study database. The edit checks include skip patterns, chronological order of events, and longitudinal edits for consistency of serial measurements using statistical procedures. Undesirable patterns of errors will be examined followed by corrective actions such as modifying data acquisition procedures, deleting data items from the acquisition, and retraining personnel. The data management team will be responsible for producing customized reports for the study. Such reports will be either static reports (e.g., reports produced on a schedule) or dynamic (e.g., reports produced on demand, such as up-to-the-minute recruitment reports). All reports will be reviewed for accuracy, consistency, and confidentiality and the reports will only be provided to researchers authorized either by the Principal Investigators or the project manager. Only authorized investigators will be able to further analyze or produce reports on selected data.

**ClinicalTrials.gov Requirements**

The study was registered in ClinicalTrials.gov, and the NCT identifier is NCT06263647. The project does not have a trial of drugs and biologics, or a trial of devices. The Principal Investigators will ensure that processes are in place to achieve and maintain compliance with the following Policy requirements for the clinical trial proposed in this application:

• Notice of changes in recruitment status will be provided as soon as possible, but no later than 30 days after such changes.

• Clinical trial summary results information will be submitted to ClinicalTrials.gov no later than 1 year following the primary completion date.

• Clinical trial information submitted to ClinicalTrials.gov will be verified, updated and corrected in accordance with all applicable deadlines established in the Final Rule for Clinical Trials Registration and Results Information Submission (42 CFR 11.64).

• Informed consent documents for FDAAA 801 Applicable Clinical Trials (ACTs) will include the statement specified at 21 CFR 50.25(c) relating to posting of clinical trial information at ClinicalTrials.gov. Informed consent documents for all other clinical trials will include a similar statement.

**Dissemination Plan:** As a part of our study design, we are excited to participate in the Community Level Interventions for Firearm Prevention Research Network through this funding mechanism and plan to share design and approach considerations, data, and programmatic considerations. This will assist in implementing and disseminating the program for future trials. After the intervention and analysis are completed, the research team will plan to submit findings to regional and national conferences in the form of abstracts and presentations. They will also compile the results for manuscript development for publications to disseminate knowledge to the greater scientific community.

**ATTACHMENTS**

1. Organizational Framework of Study Team
2. Research Timeline
3. Linking Log
4. References

**Figure 1. Study Team**


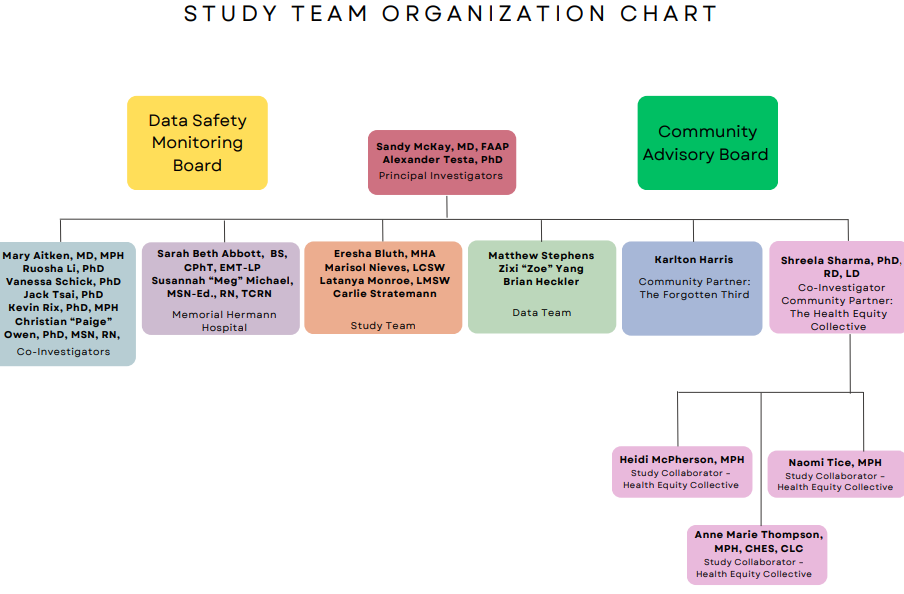


Table 1: **Research Timeline -UG3**


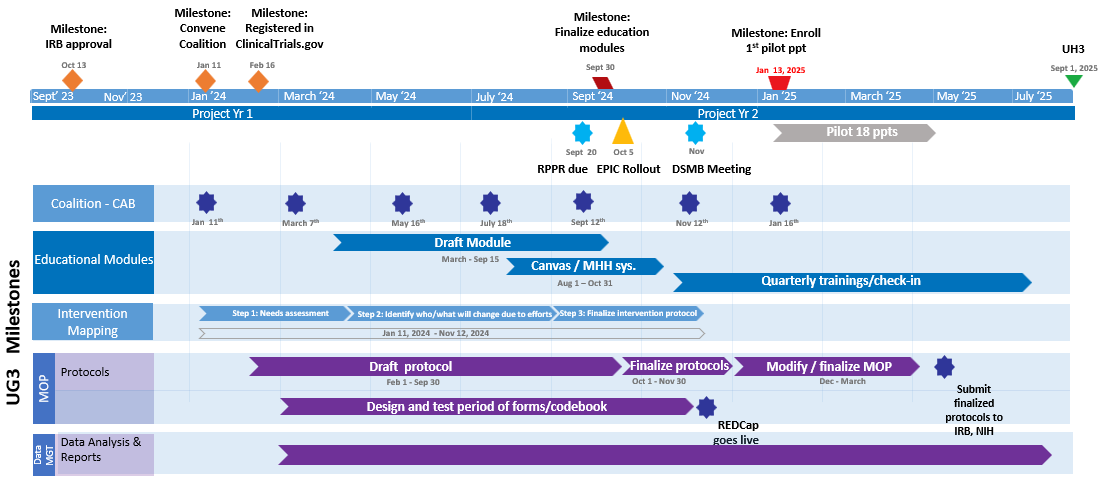


**Research Timeline -UH3**


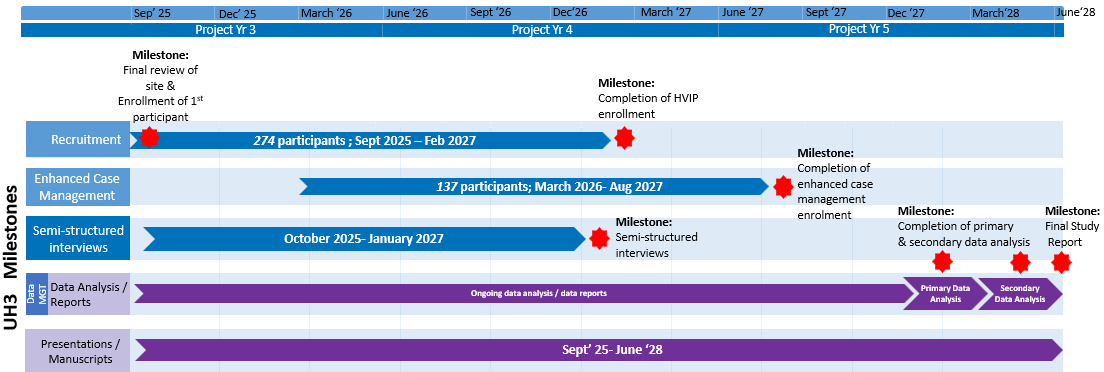


3. Table 2: Subject Linking Log

| **Study Title:** |  |
| --- | --- |
| **Principal Investigator** |  |
| **Study Coordinator:** |  |

| **Subject Name** | **Initials** | **Unique Study Identifier** | **Comments** |
| --- | --- | --- | --- |
|  |  |  |  |
|  |  |  |  |
|  |  |  |  |
|  |  |  |  |
|  |  |  |  |
|  |  |  |  |
|  |  |  |  |
|  |  |  |  |

**References**

1. Kok G, Gottlieb NH, Peters GJY, et al. A taxonomy of behaviour change methods: an Intervention Mapping approach. *Health Psychol Rev*. 2016;10(3):297-312. doi:10.1080/17437199.2015.1077155

2. Hesselink G, Zegers M, Vernooij-Dassen M, et al. Improving patient discharge and reducing hospital readmissions by using Intervention Mapping. *BMC Health Services Research*. 2014;14(1):389. doi:10.1186/1472-6963-14-389

3. Bates R. A critical analysis of evaluation practice: the Kirkpatrick model and the principle of beneficence. *Evaluation and Program Planning*. 2004;27(3):341-347. doi:10.1016/j.evalprogplan.2004.04.011

4. Heydari MR, Taghva F, Amini M, Delavari S. Using Kirkpatrick’s model to measure the effect of a new teaching and learning methods workshop for health care staff. *BMC Research Notes*. 2019;12(1):388. doi:10.1186/s13104-019-4421-y

5. Borkan J. *Immersion-Crystallization: A Valuable Analytic Tool for Healthcare Research*. Sage Publications; 2022.

6. Farmer T, Robinson K, Elliott SJ, Eyles J. Developing and Implementing a Triangulation Protocol for Qualitative Health Research. *Qual Health Res*. 2006;16(3):377-394. doi:10.1177/1049732305285708

7. Thurmond VA. The Point of Triangulation. *Journal of Nursing Scholarship*. 2001;33(3):253-258. doi:10.1111/j.1547-5069.2001.00253.x

8. Mays N, Pope C. Qualitative Research: Rigour and qualitative research. *BMJ*. 1995;311(6997):109-112. doi:10.1136/bmj.311.6997.109

9. Western B, Braga A, Hureau D, Sirois C. Study retention as bias reduction in a hard-to-reach population. *Proceedings of the National Academy of Sciences*. 2016;113(20):5477-5485. doi:10.1073/pnas.1604138113

10. Hennink M, Kaiser BN. Sample sizes for saturation in qualitative research: A systematic review of empirical tests. Soc Sci Med. 2022;292:114523. doi:10.1016/j.socscimed.2021.114523

11. Carter PM, Walton MA, Roehler DR, Goldstick J, Zimmerman MA, Blow FC, Cunningham RM. Firearm violence among high-risk emergency department youth after an assault injury. Pediatrics. 2015 May;135(5):805-15. doi: 10.1542/peds.2014-3572.
